# Divergent CD45^+^ immune landscapes shape the lung tumor microenvironment

**DOI:** 10.3389/fimmu.2026.1765833

**Published:** 2026-02-09

**Authors:** Selma Dizdarević, René Wiegandt, Andreas Weigert, Thorsten Stiewe, Bastian Eul, Stefan Guenther, Friedrich Grimminger, Werner Seeger, Soni Savai Pullamsetti, Mario Looso, Kati Turkowski, Rajkumar Savai

**Affiliations:** 1 Institute for Lung Health (ILH), Justus-Liebig University, Giessen, Germany; 2 Max-Planck Institute for Heart and Lung Research, Member of the German Center for Lung Research (DZL), Member of the Cardio-Pulmonary Institute (CPI), Bad Nauheim, Germany; 3 Department for Immunity of Inflammation, Mannheim Institute for Innate Immunoscience (MI3), Medical Faculty Mannheim, Heidelberg University, Mannheim, Germany; 4 Institute of Biochemistry I, Goethe-University Frankfurt, Frankfurt, Germany; 5 Institute of Molecular Oncology, Philipps University, Marburg, Germany; 6 Department of Internal Medicine, Justus-Liebig University Giessen, Member of the DZL, Member of CPI, Giessen, Germany; 7 Department of Medicine, Quebec Heart and Lung Institute, Laval University, Québec City, QC, Canada

**Keywords:** CD45^+^ cells, Kras^LA2^, lung cancer, single-cell RNA sequencing, tumor microenvironment

## Abstract

**Background:**

The lung tumor microenvironment (TME) plays a crucial role in the progression and metastasis of lung cancer. It consists of various cell types that interact in complex ways to influence tumor behavior. CD45^+^ cells, as a component of the TME, have complex and multifaceted roles in lung cancer. The balance between the anti-tumor and pro-tumor functions of CD45^+^ cells can significantly affect lung cancer outcomes. Understanding these roles is essential for developing targeted therapies that harness the beneficial effects of CD45^+^ cells while mitigating their harmful effects.

**Methods:**

We performed single-cell RNA sequencing of sorted CD45^+^ immune cells from healthy lungs, orthotopic LLC1 tumors, and Kras^LA2^ (Kras) genetically engineered tumors. Analyses included immune composition, transcriptional programs, differentiation trajectories, metabolic states, and ligand-receptor-based intercellular communication networks.

**Results:**

Four major immune compartments, B cells, T cells, NK cells, and macrophages, underwent model-specific remodeling. LLC1 tumors showed B cell expansion and T and NK cell reduction, with inflammatory, stress-response, and NF−κB/TNF-dominant programs. Kras^LA2^ tumors retained a balanced immune composition but exhibited metabolic rewiring, elevated antigen-presentation signatures, and selective intercellular signaling. Subclustering revealed specialized changes across B cell (resting, mature, pre-Bcr, late pro-B, plasma), T cell (Cd4^+^, Cd8^+^, memory, activated, Treg, Th17), NK cell (Fcgr3^high^, Fcgr3^low^, Xcl1^+^), and macrophage (Ace^+^, Bcr^+^, Ccr2^+^, Cd3^+^, metabolic, MHCII^+^) subsets. Ligand-receptor analyses highlighted dense inflammatory networks in LLC1 tumors versus metabolically tuned signaling in Kras^LA2^ tumors.

**Conclusion:**

Distinct CD45^+^ immune landscapes, characterized by inflammatory suppression in LLC1 and metabolic adaptation in Kras^LA2^ tumors, shape lung tumor biology. This atlas identifies genotype-specific immune vulnerabilities with potential relevance for precision immunotherapy in non-small cell lung cancer.

## 1 Introduction

Lung cancer remains the leading cause of cancer-related deaths worldwide, and a deeper understanding of the tumor microenvironment (TME) is essential for developing more effective therapies. The TME includes diverse cellular components such as immune, endothelial, and stromal cells, as well as non-cellular components like the extracellular matrix (ECM) and soluble signaling factors (1–4). All these components play important roles in tumor initiation, progression, metastasis, and therapeutic response. Current therapeutic approaches target various components of the lung TME to impede cancer progression. Precise characterization of the cell populations within the TME could facilitate the development of novel and effective targeted therapies. The heterogeneity of CD45^+^ cells in the lung cancer TME is of particular interest due to their dual capacity to suppress or promote tumor growth, depending on their phenotype, abundance, and spatial distribution. Interactions between these immune cells and tumor-derived factors further influence disease behavior. For example, tumor-derived exosomes have been shown to transfer microRNAs to these cells, activating pathways that promote tumor progression. Studies on miR-21 and miR-29a have shown that these microRNAs can enhance Toll-like receptor (TLR) signaling and contribute to immune modulation and cancer metastasis (5).

In non-small cell lung cancer (NSCLC), the compositional dynamics of CD45^+^ cells within the TME identified at least thirteen distinct immune cell types, with a dominant presence of Cd4^+^ T cells and Cd8^+^ T cells highlighting a robust adaptive immune response aimed at tumor elimination (6). Furthermore, studies using single-cell RNA sequencing (scRNA-seq) are providing increasingly deeper insights into the functional states and interactions of different immune cells within the TME. For example, T regulatory (Treg) cells and exhausted Cd8^+^ T cells are prevalent in both early and metastatic lung cancer, acting as mediators of immune suppression that can facilitate tumor progression (7). Another study identified three immunosuppressive clusters: exhausted Cd8^+^ T cells, pro-inflammatory M2 tumor-associated macrophages, and tumor-promoting regulatory B cells, which collectively suppress anti-tumor immunity and promote tumor progression (8). Specific immune cell types may have different effects on tumor dynamics depending on the cancer subtype, further complicating the understanding of immune interactions within the TME. A study of 72,475 immune cells from lung cancer patients revealed distinct immune compositions and gene expression profiles for each subtype. CD45^+^ cells included diverse populations in tumors and adjacent tissues. Lung adenocarcinoma had more myeloid cells with immune regulatory and lipid metabolism signatures, while squamous cell carcinoma had increased cytotoxic and effector T and NK cells associated with T cell activation and cytokine responses, highlighting key subsets that shape the tumor immune microenvironment (9). CD45^+^ immune cells within the lung cancer TME are highly heterogeneous, consisting of diverse subtypes with distinct functional states that differ by lung cancer subtype. This heterogeneity has important implications for tumor progression and therapeutic responses, making it a key focus of research to improve immunotherapeutic strategies.

To overcome the limitations of bulk and low-resolution approaches, we performed scRNA-seq of CD45^+^ immune cells from healthy lungs, orthotopic tumors, and Kras^LA2^ (Kras) mouse models. This analysis revealed extensive immune remodeling and uncovered both known and previously unrecognized macrophage, T cell, B cell, and NK cell subsets. Distinct transcriptional programs reflected tumor-specific immune adaptation and functional diversity. Together, these findings provide a high-resolution map of the lung TME and highlight cellular and molecular pathways that may inform future immunotherapeutic strategies.

## 2 Methods

### 2.1 Animal experiments

All animal experiments were approved by the local authorities (Regierungspräsidium Darmstadt, Hessen, Germany) and conducted in accordance with European Union guidelines for the care and use of laboratory animals. Mice were housed under specific pathogen-free conditions with a 12-hour light/dark cycle, controlled humidity (30–70%) and temperature (20–26°C), and had ad libitum access to water and standard chow. Two murine lung cancer models were used in this study: (i) an intravenous lung tumor model, in which LLC1 cells (1 × 10^6^) were injected into C57BL/6 mice via the tail vein and animals were closely monitored for up to 18 days after injection (10), and (ii) a Kras^LA2^ genetically engineered mouse model of lung adenocarcinoma, genotyped according to The Jackson Laboratory–recommended protocol (11). Mice were euthanized upon reaching predefined humane endpoints, and lungs were harvested and processed for fluorescence-activated cell sorting (FACS) analysis.

### 2.2 Sample preparation and single-cell RNA sequencing

For CD45^+^ cell isolation, lungs from three mice per condition (C57BL/6J controls, LLC1 tumor-bearing, and Kras^LA2^ tumor-bearing) were processed individually. Each lung was minced into small pieces and digested in buffer containing 5% collagenase and 1% DNase I for 30 minutes at 37°C. After enzymatic digestion, cell suspensions were passed sequentially through a 100 µm cell strainer and a 40 µm nylon mesh. Cells were collected by centrifugation for 10 minutes at 500 × g and resuspended in 2 ml erythrocyte lysis buffer (Erylysis) to remove red blood cells. After 4 minutes of incubation, lysis was quenched by adding 23 ml PBS. Cells were then washed, resuspended in PBS, and sorted for CD45^+^ cells using a BD FACSAria III cell sorter. Dead cells were excluded based on 7-AAD staining as indicated in the gating strategy (**Supplementary Figures S1A, B**). Following FACS, the sorted CD45^+^ fractions from the three mice of each condition were pooled to generate one combined sample per condition. The cell suspensions were counted with a Moxi cell counter and diluted according to the manufacturer’s protocol to obtain 10,000 single-cell data points per sample. Each sample was run separately on a lane in the Chromium Controller with Chromium Next GEM Single Cell 3′ Reagent Kits v3.1 (10x Genomics). Single-cell RNA seq library preparation was performed using the standard protocol. Sequencing was performed on a NextSeq 500, and raw reads were aligned to the mouse genome (mm10) and counted by StarSolo (12), followed by secondary analysis in Annotated Data Format.

Preprocessed counts were further analyzed using Scanpy (13). Basic cell quality control was performed by considering the number of detected genes and mitochondrial content. We removed 21 cells that expressed fewer than 300 genes or had mitochondrial content greater than 8%. Additionally, we filtered out 14,083 genes detected in fewer than 30 cells (<0.01%). All ribosomal and sex-associated genes were excluded from the dataset. Raw counts per cell were normalized to the median count across all cells and transformed into log space to stabilize variance. We initially reduced the dimensionality of the dataset using PCA, retaining 50 principal components.

### 2.3 Cell clustering

All downstream analyses were performed with the SC-Framework (14) (version 0.13.0b). Based on the first 6 PCs from the initial PCA, the data were transformed into a low-dimensional embedding using Uniform Manifold Approximation and Projection (UMAP) (15) (spread = 2.0, min_dist = 0.3). Clustering was performed using the Leiden algorithm (16) with a resolution of 0.5. Further refinement yielded 4 final clusters. Marker genes, defined as genes predominantly expressed within a specific cluster or group, were identified using the “rank_genes_groups” function in Scanpy (13). This method applies a t-test (17) with multiple hypothesis testing correction using the Benjamini-Hochberg procedure to control the false discovery rate (18). Markers were subsequently filtered based on the following criteria: (i) at least 25% of cells within the respective cluster expressing the marker gene, (ii) a fold change ≥ 0.5 compared to the aggregate expression of all other clusters, and (iii) expression ≤ 80% outside the group. Condition-specific markers were identified by applying the same function and filter independently to each subset stratified by condition. Cluster annotation was performed manually by comparing identified marker genes with publicly accessible databases. Subsets were generated from each cluster and subjected to subclustering, with all downstream analyses consistently performed on each resulting subset.

### 2.4 Pathway enrichment analysis

For the pathway analysis, cluster-specific and condition-specific marker genes were used separately as preranked inputs for KOBAS 2.0 (19), utilizing the KEGG Pathway reference database. Two separate tests were performed using either upregulated or downregulated genes to identify perturbed pathways in each direction. The results were integrated by retaining pathways that showed significant overrepresentation with a Benjamini-Hochberg adjusted p-value < 0.2 in only one input dataset, indicating pathways distinctly enriched for either upregulated or downregulated genes, but not both. The top 20 pathways were selected for each contrast and direction of regulation.

### 2.5 Receptor-ligand analysis

Receptor-ligand interactions were calculated using the z-score of the group mean expression, scaled by group size and weighted by the proportion of cells expressing the respective gene. The interaction score was defined as the sum of valid receptor-ligand pairings, based on the “mouseconsensus” database from the LIANA package (20). Differential analysis of receptor-ligand interactions between conditions was performed by calculating pairwise quantile-ranked differences of the interaction scores.

### 2.6 Flow cytometric validation of Cd3^+^ macrophages

Single-cell suspensions were prepared from Kras^LA2^ lungs (n=4) as described above. Cells were first blocked with Fc receptor blocking solution (Miltenyi Biotec) to reduce nonspecific binding. Viable cells were identified using propidium iodide (PI) exclusion. Cells were then stained with the following Miltenyi Biotec antibodies: CD45 (REA737), CD3 (REA641), and F4/80 (REA126). Appropriate isotype controls and fluorescence-minus-one (FMO) controls were included to set gates and assess nonspecific staining. Stained cells were analyzed on a flow cytometer (MACSQuant Analyzer 16, Miltenyi Biotec). For quantification of CD3 expression in macrophages, mean fluorescence intensity (MFI) was measured. Data were analyzed using FlowJo software (version 10.10.0).

### 2.7 Validation of Cd3^+^ macrophages by immunofluorescence staining

Immunofluorescence of formalin-fixed, paraffin-embedded (FFPE) Kras lung tumor tissue was performed using the PhenoCycler-Fusion platform (Akoya Biosciences). Three-micrometer-thick sections were mounted onto charged glass slides and baked at 65 °C for one hour. Slides were deparaffinized in xylene and rehydrated through graded alcohols to distilled water. Heat-induced epitope retrieval was carried out in DAKO PT Link antigen retrieval buffer (pH 9) at high pressure for 20 minutes, followed by cooling to room temperature. The slides were rinsed in ddH_2_O, washed for two minutes, and then washed twice with hydration buffer for two minutes each, followed by an additional 30 minutes with staining buffer. Primary antibodies against mouse CD3ϵ (T lymphocytes) and F4/80 (macrophages) were used. Antibodies were either obtained pre-conjugated or conjugated to unique oligonucleotide barcodes using the Akoya antibody-oligonucleotide conjugation chemistry following the manufacturer’s protocol. Conjugation efficiency and staining specificity were verified on control mouse spleen and lung tissue prior to experimental acquisition. Slides were incubated with the antibody cocktail overnight at 4°C in a humidified chamber and subsequently washed in PBST. Sections were fixed with 1.6% PFA, methanol and fixative reagent to stabilize antibody-oligonucleotide complexes according to manufactures instructions. Stained slides were loaded onto the PhenoCycler-Fusion instrument, and automated reporter hybridization, imaging, and cleavage cycles were performed according to the manufacturer’s recommendations. Fluorescent reporters complementary to CD3ϵ- and F4/80-specific barcodes were hybridized sequentially and imaged. Nuclear staining with 4′,6-diamidino-2-phenylindole (DAPI) was used for image registration and cell segmentation. Images were acquired using a 20× objective, with exposure times optimized for each channel to avoid saturation. Tile scanning and stitching were performed using PhenoCycler-Fusion software. Instrument-provided quality control metrics were monitored for signal-to-noise ratio, tissue integrity, and cycle registration accuracy.

### 2.8 Statistics

Proportion Analysis: Relative variations in cell populations across different conditions were assessed using Scanpro (21). Proportional data were logit-transformed to facilitate statistical analysis. Statistical analyses were performed using GraphPad Prism 9 software (GraphPad Inc., San Diego, CA, USA). Unpaired t-tests were used to compare means between two independent groups.

## 3 Results

### 3.1 Lung tumor type dictates immune landscape, transcriptional programs, and cellular crosstalk

**Figure 1:**
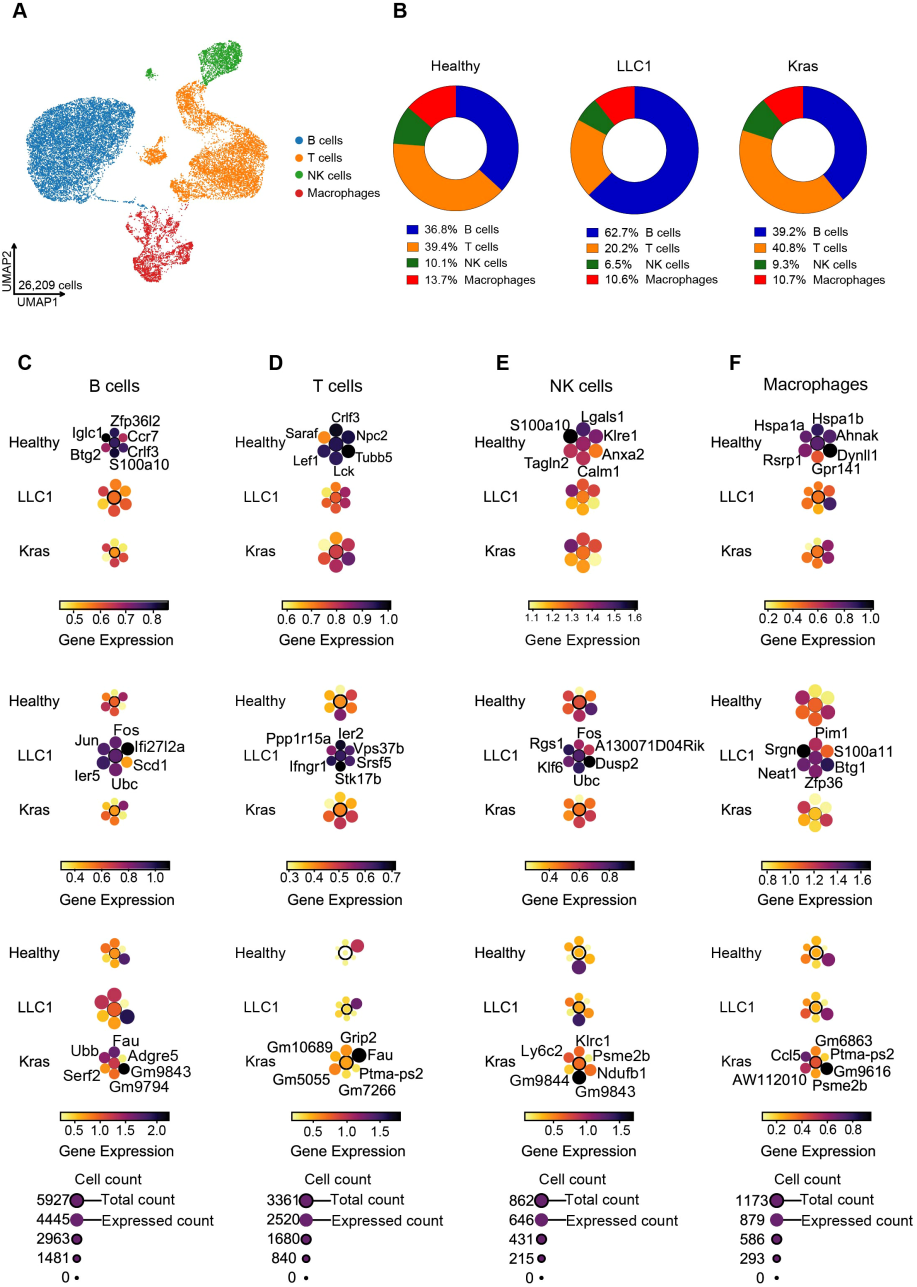
UMAP visualization of single-cell transcriptomes from CD45^+^ cells in healthy and lung cancer models showing four distinct immune populations with altered proportions and marker gene expression in tumor-bearing lungs. **(A)** Schematic of experimental workflow showing isolation from single cell suspension of mice lungs (healthy, orthotopic (LLC1) and oncogenic model (Kras^LA2^)). **(B)** Pie chart representing the percentage of clusters within the CD45^+^ cell population for all three models (healthy, LLC1 and Kras^LA2^). **(C–F)** Planet plots of B cells **(C)**, T cells **(D)**, NK cells **(E)**, and macrophages **(F)** illustrating 6 distinct marker genes in healthy, LLC1 and Kras^LA2^ mouse models, featuring a central circle representing aggregated gene set expression and surrounding circles depicting individual gene expression levels.

To investigate immune heterogeneity, scRNA-seq was performed on CD45^+^ immune cells from healthy mice, LLC1, and oncogenic Kras^LA2^ lung tumors. UMAP-based clustering identified four major immune populations: B cells, T cells, NK cells, and macrophages (**Figure 1A**), as determined by the expression of canonical marker genes in all three models (**Supplementary Figures S2A**). Notably, the composition of immune cell populations varied between models. In healthy lungs, the CD45^+^ compartment consisted of 36.8% B cells, 39.4% T cells, 10.1% NK cells, and 13.7% macrophages. LLC1 tumors induced dramatic changes, with B cells expanding to 62.7%, while T cells and NK cells decreased to 20.2% and 6.5%, respectively, and macrophages remained relatively stable at 10.6%. Kras^LA2^-driven tumors maintained a composition closer to healthy lungs, with 39.2% B cells, 40.8% T cells, 9.3% NK cells, and 10.7% macrophages (**Figure 1B**). Further analysis of the proportions of cell types between the models revealed a significant increase in B cells and a decrease in T cells in the LLC1 model compared to healthy and Kras^LA2^ mice (**Supplementary Figure S2B**). These results indicate that LLC1 tumors preferentially reshape adaptive immune populations, while Kras^LA2^ tumors maintain a more balanced immune landscape. B cell transcriptional programs were model-specific, reflecting the observed proportional changes. LLC1 B cells upregulated *Ier5, Jun, Fos, Ifi27l2a*, and *Scd1*, consistent with activation, inflammation, and metabolic stress. In contrast, Kras^LA2^ B cells expressed *Fau, Ubb, Serf2*, and *Adgre5*, associated with stress responses and adhesion, whereas healthy B cells maintained homeostasis genes (*Zfp36l2, Iglc1, Ccr7, Btg2*) (**Figures 1C**; **Supplementary Figure S2C**). T cells also displayed context-dependent states. In healthy lungs, T cells expressed *Crlf3, Lck, Lef1*, and *Saraf*, reflecting normal activation. LLC1 T cells upregulated *Ier2, Stk17b, Ifngr1*, and *Ppp1r15a*, indicating an activated, potentially exhausted phenotype. Kras^LA2^ T cells expressed *Grip2, Fau*, and *Ptmaps2*, showing a distinct activation state different from both healthy and LLC1 lungs (**Figures 1D**; **Supplementary Figure S2D**). NK cells followed similar patterns: healthy NK cells expressed *Lgals1, S100a10, Klre1, Anxa2*, and *Tagln2* (homeostatic surveillance); LLC1 NK cells upregulated *Fos, Dusp2, Klf6*, and *Ubc* (activation and stress); and Kras^LA2^ NK cells expressed *Klrc1, Psme2b*, and *Ly6c2*, suggesting functional suppression (**Figures 1E**; **Supplementary Figure S2E**). Macrophages exhibited relative compositional stability but context-dependent transcriptional states. Healthy macrophages expressed *Hspa1a, Hspa1b, Ahnak, Dynll1*, and *Gpr141*, while LLC1 macrophages upregulated *Pim1, S100a11*, and *Neat1* (pro-inflammatory, tumor-associated), and Kras^LA2^ macrophages expressed *Ccl5* and *Psme2b*, indicative of immunomodulatory and antigen-presenting roles (**Figures 1F**; **Supplementary Figure S2F**).

**Figure 2:**
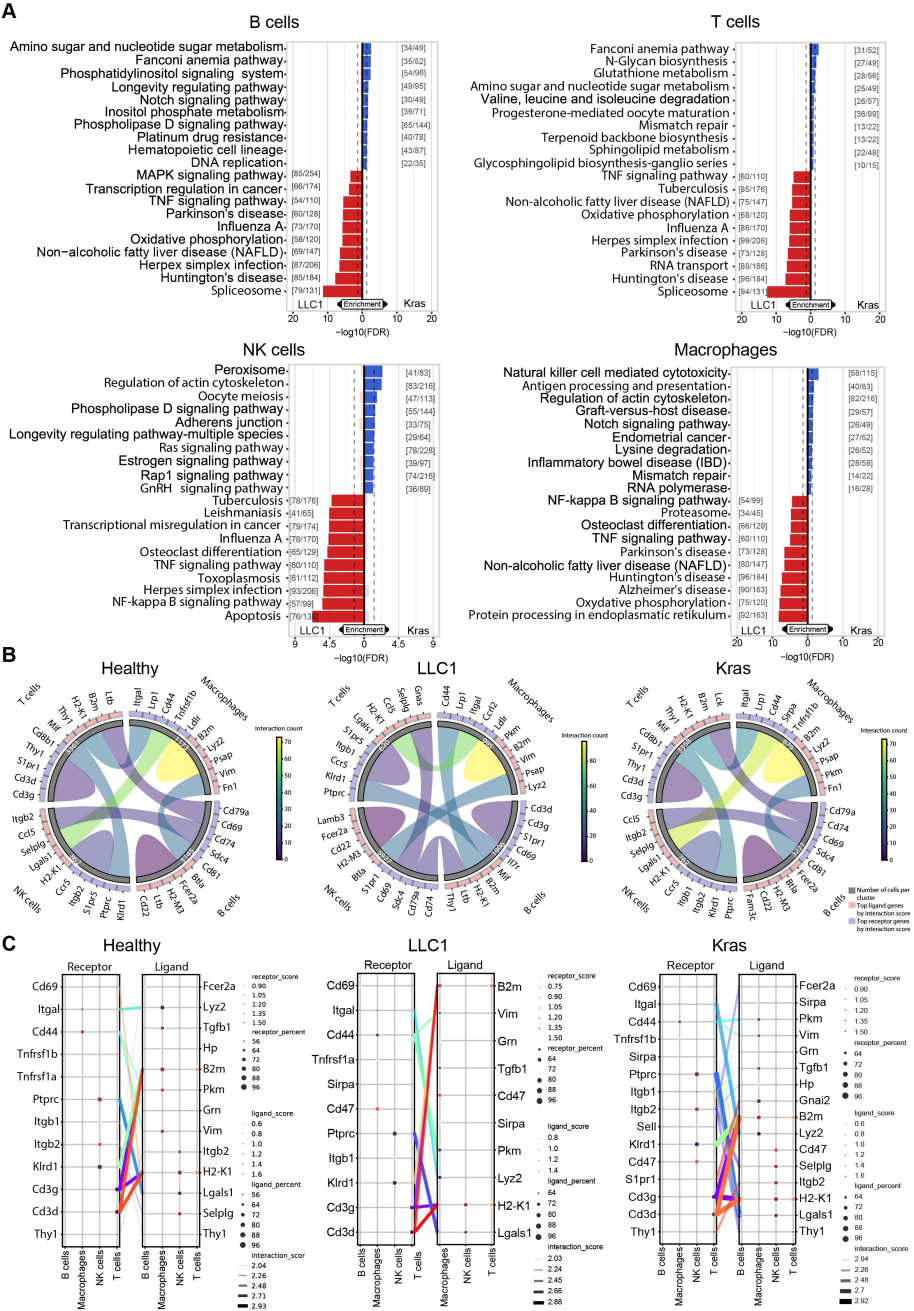
GSEA reveal function specialization of immune subsets in mouse model depend manner. **(A)** Pathway enrichment analysis using the KEGG database highlights the upregulated pathways in LLC1 versus Kras^LA2^ mouse models for each cell cluster (B cells, T cells, NK cells and macrophages). **(B)** Cyclone plots depict the overall communication patterns between different cell cluster (B cells, T cells, NK cells, and macrophages). The top five receptors and ligands for each cluster are emphasized in the visualization. **(C)** Connection plots illustrate the most significant ligand-receptor interactions between clusters across lung cancer models (healthy, LLC1 and Kras^LA2^) by showing distinct receptor-ligand pairs that are enriched (receptor/ligand score) with a certain cell coverage (receptor/ligand percent).

Gene set enrichment analysis highlighted the functional specialization of immune subsets. In B cells, LLC1 tumors activated MAPK and TNF signaling, while Kras^LA2^ B cells enriched metabolic pathways, including inositol phosphate metabolism and Notch signaling. LLC1 macrophages upregulated NF−κB signaling, oxidative phosphorylation, and endoplasmic reticulum (ER) protein processing, whereas Kras^LA2^ macrophages enriched NK cell-mediated cytotoxicity and antigen presentation pathways. T cells in LLC1 tumors showed oxidative phosphorylation, TNF signaling, and spliceosome activation, while Kras^LA2^ T cells activated glutathione metabolism, N-glycan biosynthesis, and amino sugar metabolism. NK cells in LLC1 tumors enriched peroxisome, Ras, and phospholipase D signaling, whereas Kras^LA2^ NK cells exhibited transcriptional dysregulation, TNF signaling, and apoptosis (**Figure 2A**). Receptor-ligand analysis revealed tumor-specific rewiring of immune interactions. NK cells and macrophages showed the highest interaction density, strongest in the Kras^LA2^ model (**Figure 2B**). Healthy lungs displayed interactions via *Cd44, Ptprc*, and *Klrd1*, while LLC1 tumors showed reduced crosstalk with decreased macrophage ligands (*Lyz2, Vim, Tgfb1*). Kras^LA2^ tumors exhibited increased interactions, including *Thy1-Itgb2* (T cell-NK cell), *Cd44-Pkm* (macrophage), and *S1pr1-Gnai2* (T cell-macrophage), highlighting context-dependent immune communication (**Figure 2C**). These findings demonstrate that the TME shapes both the composition and functional states of lung immune cells in a model-dependent manner. LLC1 tumors drive B cell expansion and adaptive immune suppression, coupled with inflammatory and stress-related transcriptional programs. Kras^LA2^-driven tumors preserve a more balanced immune composition but induce distinct transcriptional remodeling and novel intercellular communication networks, emphasizing context-specific immune regulation and functional specialization across B cells, T cells, NK cells, and macrophages.

### 3.2 Tumor-driven expansion and functional divergence of antigen-presenting and regulatory B cells

**Figure 3:**
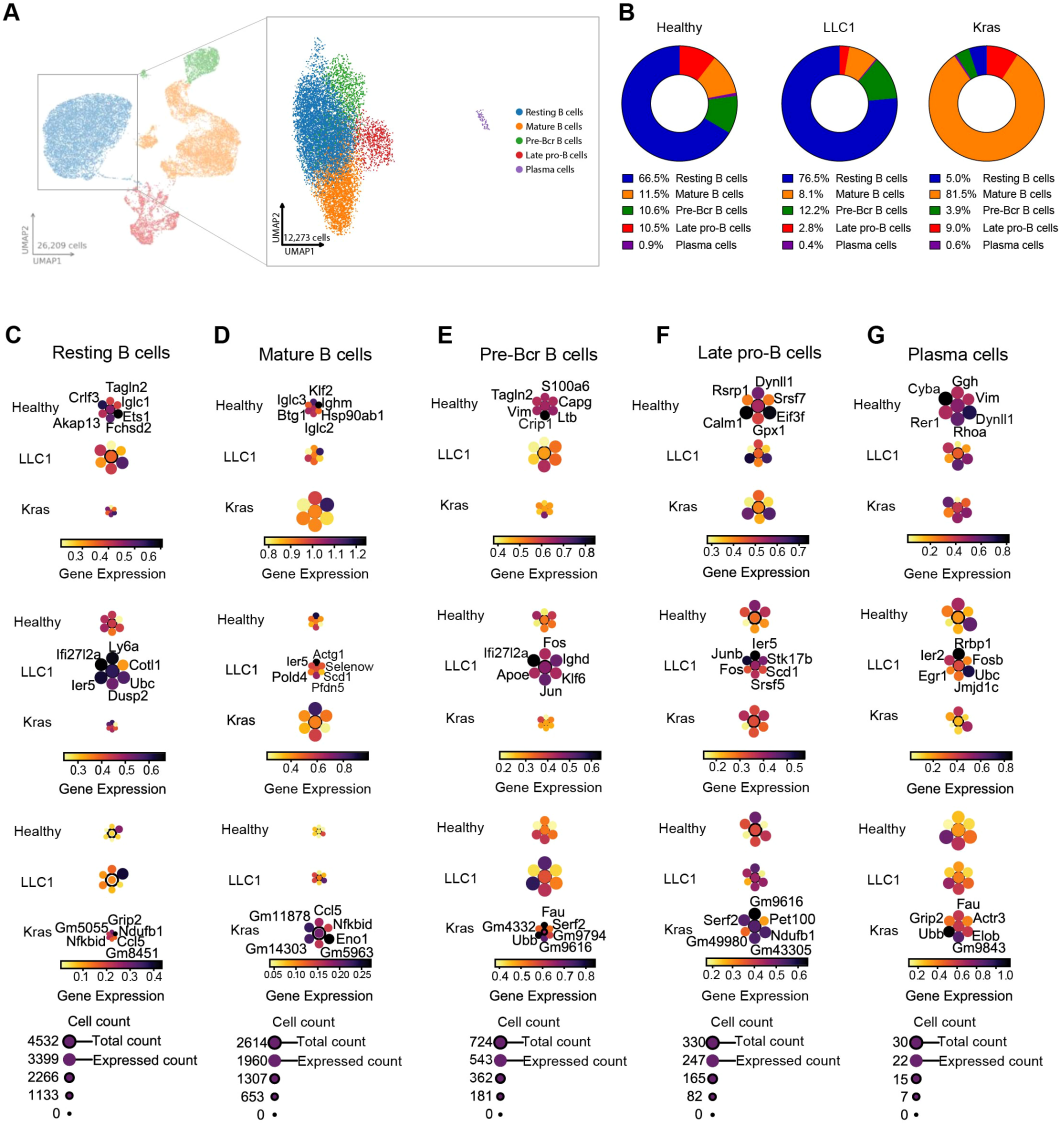
Tumor-driven expansion and functional divergence of antigen-presenting and regulatory B cells. **(A)** UMAP plots illustrate major immune cell compartments identified among CD45^+^ cells (left), and refined subcluster annotations within the B cell compartment (right), including resting B cells, mature B cells, pre-Bcr B cells, late pro-B cells, and plasma cells. **(B)** Pie chart representing the percentage of B cell subclusters within the CD45^+^ cell population for all three models (healthy, LLC1 and Kras^LA2^). **(C–G)** Planet plots of resting B cells **(C)**, mature B cells **(D)**, pre-Bcr B cells **(E)**, late pro-B cells **(F)** and plasma cells **(G)** illustrating 6 distinct marker genes in healthy, LLC1 and Kras^LA2^ mouse models, featuring a central circle representing aggregated gene expression and surrounding circles depicting individual gene expression levels.

**Figure 4:**
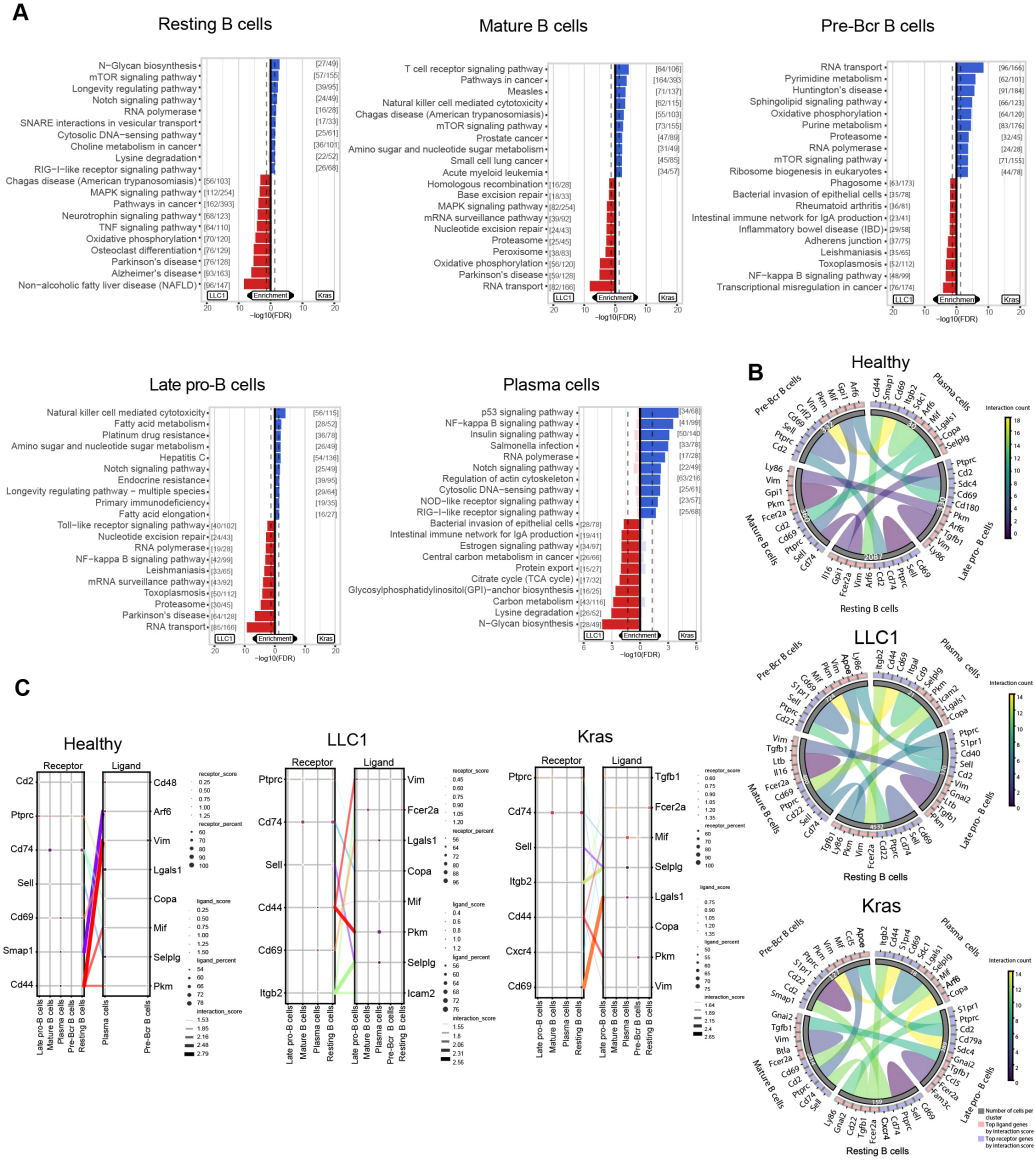
Shifts in B cell proportions correspond to tumor-specific transcriptional programs and ligand-receptor interactions. **(A)** Pathway enrichment analysis using the KEGG database highlights upregulated pathways in LLC1 compared to Kras^LA2^ mouse models for each cell subcluster (resting B cells, mature B cells, pre-Bcr B cells, late pro-B cells, and plasma cells). **(B)** Cyclone plots show overall communication patterns between different cell subclusters. The top five receptors and ligands for each cluster are highlighted in the visualization. **(C)** Connection plots display the most significant ligand-receptor interactions between clusters across lung cancer models (healthy, LLC1, and Kras^LA2^) by showing distinct receptor-ligand pairs that are enriched (receptor/ligand score) with a certain cell coverage (receptor/ligand percent).

B cells constituted one of the largest immune clusters in healthy, LLC1, and Kras^LA2^ tumor-bearing mice. Subclustering based on canonical marker genes identified transcriptionally distinct subsets: late pro-B cells, mature B cells, plasma cells, pre-Bcr cells, and resting B cells (**Figures 3A**; **Supplementary Figure S3A**). The proportions of these subsets varied markedly across models (**Figures 3B**; **Supplementary Figure S3B**). Mature B cells were the predominant subset in Kras^LA2^ tumors (81.5%) but represented minor populations in healthy (11.5%) and LLC1 (8.1%) lungs. In contrast, resting B cells comprised the majority in healthy (66.4%) and LLC1 (76.5%) mice but were rare in Kras^LA2^ (5%). Pre-Bcr cells were also reduced in Kras^LA2^ (3.9%) compared to healthy (10.6%) and LLC1 (12.2%), while late pro-B cells were lower in LLC1 (2.8%) than in healthy (10.5%) and Kras^LA2^ (9%). Plasma cells were consistently rare (<1%) across all models. These shifts suggest that Kras^LA2^ tumors drive expansion of mature B cells at the expense of resting and early B cell populations, whereas LLC1 maintains a more homeostatic B cell composition (**Figures 3B**; **Supplementary Figure S3B**). Transcriptional programs reflected these proportional changes. Resting B cells in Kras^LA2^, though numerically limited, enriched immune signaling and metabolism genes (*Grip2, Nfkbid, Ccl5, Ndufb1*), whereas LLC1 and healthy resting B cells maintained homeostatic or stress-response programs (**Figures 3C**; **Supplementary Figure S3C**). Kras^LA2^ mature B cells upregulated pro-inflammatory and immunomodulatory genes (*Ccl5, Nfkbid, Eno1*), consistent with their dominance in the TME, while LLC1 mature B cells expressed cytoskeletal, stress, and metabolic genes (*Actg1, Ier5, Scd1, Selenow*) (**Figures 3D**; **Supplementary Figure S3D**). Late pro-B and pre-Bcr cells exhibited tumor-specific transcriptional adaptations, with Kras^LA2^ subsets favoring mitochondrial function and protein turnover (*Serf2, Fau, Ubb*) and LLC1 subsets expressing stress and differentiation genes (*Ier5, Fos, Jun*) (**Figures 3E, F**; **Supplementary Figures S3E, F**). Plasma cells, despite their low abundance, displayed model-specific gene signatures: Kras^LA2^ cells upregulated ubiquitination and cytoskeletal remodeling genes, while LLC1 cells activated stress-response and immediate-early genes (**Figures 3G**; **Supplementary Figure S3G**). Pathway and receptor-ligand analyses further reflected these differences. Kras^LA2^ B cells enriched NK cell-mediated cytotoxicity, mTOR, TCR, and N-glycan biosynthesis pathways, whereas LLC1 subsets activated NF−κB, Toll-like receptor, MAPK, and oxidative phosphorylation pathways (**Figure 4A**). Kras^LA2^ B cells also exhibited resting-to-plasma cell crosstalk (*Cd69-Lgals1, Sell-Selplg*), while LLC1 tumors displayed extensive cross-subcluster interactions and healthy lungs showed primarily plasma cell self-interactions (**Figures 4B, C**). Overall, shifts in B cell proportions align with tumor-specific transcriptional programs and intercellular communication patterns, highlighting the expansion of functionally active mature B cells in Kras^LA2^ tumors and the maintenance of homeostatic populations in LLC1 and healthy lungs.

### 3.3 Context-dependent reprogramming of Cd4^+^, Cd8^+^, and regulatory T cells in lung tumors

**Figure 5:**
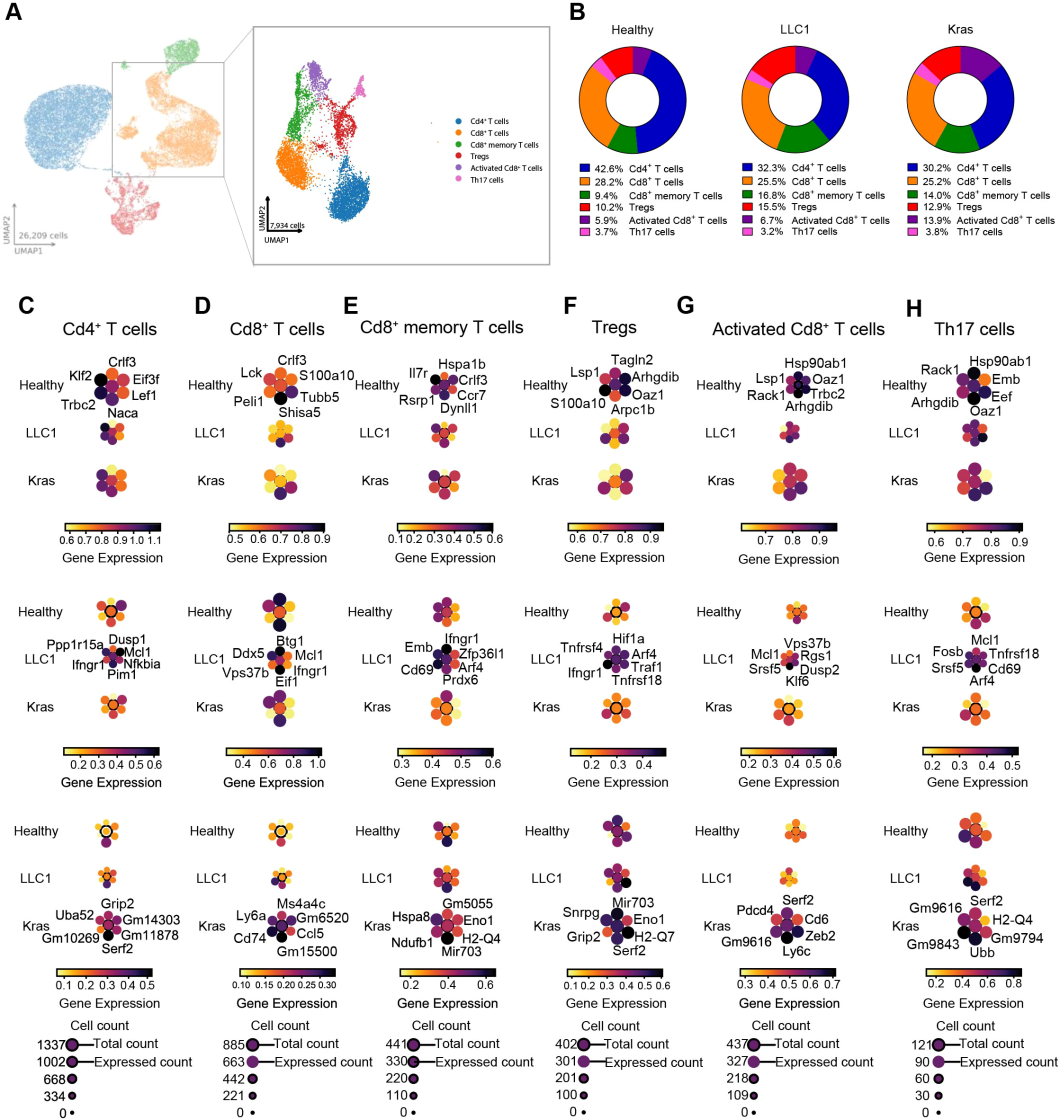
Remodeling of Cd4^+^, Cd8^+^, and Treg cells with distinct transcriptional and functional programs. **(A)** UMAP plots show major immune cell compartments identified among CD45^+^ cells (left) and refined subcluster annotations within the T cell compartment (right), including Cd4^+^ T cells, Cd8^+^ T cells, Cd8^+^ memory T cells, Tregs, activated Cd8^+^ T cells, and Th17 cells. **(B)** Pie chart showing the percentage of T cell subsets within the CD45^+^ cell population for all three models (healthy, LLC1, and Kras^LA2^). **(C–H)** Planet plots of resting Cd4^+^ T cells **(C)**, Cd8^+^ T cells **(D)**, Cd8^+^ memory T cells **(E)**, Tregs **(F)**, activated Cd8^+^ T cells **(G)**, and Th17 cells **(H)** illustrating six distinct marker genes in healthy, LLC1, and Kras^LA2^ mouse models, with a central circle representing aggregated gene expression and surrounding circles showing individual gene expression levels.

After analyzing the B cell compartment, we next examined the T cell population, a critical component of the immune landscape in all conditions. Given the pivotal role of T cells in anti-tumor immunity, we assessed their heterogeneity in greater detail. Subclustering based on canonical marker gene expression revealed six distinct T cell subpopulations across healthy, LLC1, and Kras^LA2^ models: activated Cd8^+^ T cells, Cd4^+^ T cells, Cd8^+^ memory T cells, Cd8^+^ T cells, T helper 17 (Th17) cells, and regulatory T cells (Tregs) (**Figures 5A**; **Supplementary Figure S4A**). Population distributions varied significantly between conditions. Cd4^+^ T cells were the most abundant subtype, representing 42.6% of T cells in healthy controls but decreasing to 32.2% and 30.2% in LLC1 and Kras^LA2^ tumors, respectively. Cd8^+^ T cells maintained a relatively stable presence across conditions (healthy: 28.2%; LLC1: 25.5%; Kras^LA2^: 25.2%). Notably, Cd8^+^ memory T cells expanded in tumor models (LLC1: 16.8%; Kras^LA2^: 14%) compared to healthy tissue (9.4%), reflecting potential antigen-driven memory responses. Activated Cd8^+^ T cells were significantly enriched in Kras^LA2^ tumors (13.9%), more than doubling their frequency relative to LLC1 (6.7%) and healthy samples (5.9%). Tregs also increased in both tumor contexts (LLC1: 15.5%; Kras^LA2^: 12.9%) compared to healthy controls (10.2%), whereas Th17 cell proportions remained stable across all groups (**Figure 5B**). However, analysis of the proportions of the different subtypes revealed no significant change between the models (**Supplementary Figure S4B**). To elucidate functional states within these subsets, we examined transcriptional profiles. Cd4^+^ T cells in Kras^LA2^ tumors upregulated genes such as *Grip2, Uba52*, and uncharacterized genes (*Gm10269, Gm11878, Gm14303*). LLC1 Cd4^+^ T cells expressed *Dusp1, Ifngr1, Pim1, Nfkbia*, and *Mcl1*, consistent with enhanced activation, apoptosis resistance, and interferon signaling. Healthy Cd4^+^ cells upregulated quiescence-associated genes *Klf2* and *Lef1* (**Figures 5C**; **Supplementary Figure S5A**). Cd8^+^ T cells exhibited model-specific adaptations: Kras^LA2^ tumors showed increased *Ms4a4c, Ly6a, Cd74*, and *Ccl5*, indicating enhanced activation, antigen presentation, and chemokine signaling. LLC1 cells upregulated *Btg1, Ddx5*, *Vps37b, Ifngr1*, and *Mcl1*, reflecting proliferation, interferon responses, and apoptosis resistance. Healthy Cd8^+^ cells expressed baseline immune signaling markers such as *Crlf3, Lck, Peli1*, and *S100a10* (**Figures 5D**; **Supplementary Figure S5B**). Cd8^+^ memory T cells showed distinct metabolic and activation signatures: Kras^LA2^-associated memory cells upregulated stress and metabolic genes (*Hspa8, Ndufb1, Eno1*), while LLC1 memory cells expressed *Ifngr1* and *Cd69*, consistent with immune engagement. Healthy memory T cells elevated *Il7r, Ccr7*, and *Hspa1b*, supporting homeostasis and migration (**Figures 5E**; **Supplementary Figure S5C**). Tregs in tumor-bearing mice exhibited immunosuppressive transcriptional programs. Kras^LA2^ Tregs upregulated *Mir703, Snrpg, Grip2, Serf2, H2-Q7*, and *Eno1*, implicating metabolic and antigen-processing adaptations. LLC1 Tregs showed increased expression of *Hif1a, Tnfrsf4* (*OX40*), *Ifngr1*, *Tnfrsf18 (GITR), Traf1*, and *Arf4*, supporting survival, activation, and immunosuppression. Healthy Tregs favored genes linked to cytoskeletal organization and homeostasis, including *Tagln2, Lsp1*, and *S100a10* (**Figures 5F**; **Supplementary Figure S5D**). Activated Cd8^+^ T cells, notably expanded in Kras^LA2^ tumors, expressed effector differentiation markers (*Zeb2, Pdcd4, Ly6c2, Cd6*) alongside exhaustion-related genes. LLC1 activated Cd8^+^ T cells upregulated *Mcl1, Dusp2, Klf6*, and *Rgs1*, indicative of anti-apoptotic mechanisms and dysfunction within an immunosuppressive microenvironment. Healthy activated Cd8^+^ cells expressed homeostatic genes *Hsp90ab1* and *Lsp1* (**Figures 5G**; **Supplementary Figure S5E**). Lastly, Th17 cells, despite stable abundance, showed model-specific transcriptional profiles. Kras^LA2^ Th17 cells upregulated *Serf2, Ubb*, and uncharacterized genes, suggesting stress response and antigen presentation roles. LLC1 Th17 cells expressed *Mcl1, Fosb, Cd69*, and *Tnfrsf18*, reflecting tumor-promoting inflammation and survival. Healthy Th17 cells favored homeostatic genes such as *Hsp90ab1* and *Rack1* (**Figures 5H**; **Supplementary Figure S5F**).

**Figure 6:**
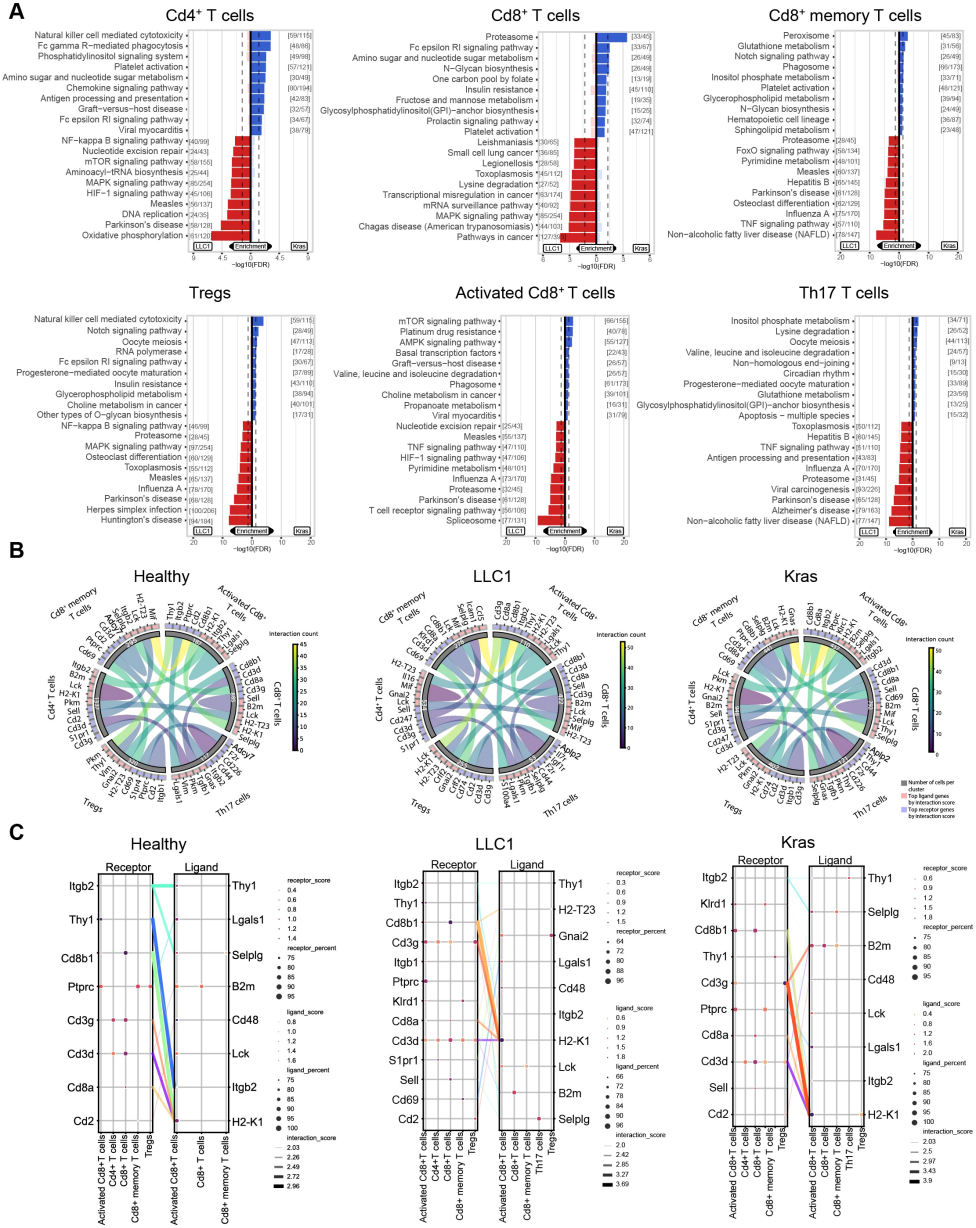
LLC1 and Kras^LA2^ lung tumors engage distinct T cell–driven immune mechanisms. **(A)** Pathway enrichment analysis using the KEGG database highlights upregulated pathways in LLC1 compared to Kras^LA2^ mouse models for each cell subcluster (Cd4^+^ T cells, Cd8^+^ T cells, Cd8^+^ memory T cells, Tregs, activated Cd8^+^ T cells, and Th17 cells). **(B)** Cyclone plots show the overall communication patterns between different cell subclusters, with the top five receptors and ligands for each cluster highlighted. **(C)** Connection plots illustrate the most significant ligand-receptor interactions between clusters across lung cancer models (healthy, LLC1, and Kras^LA2^) by displaying distinct receptor-ligand pairs that are enriched (receptor/ligand score) with a specific cell coverage (receptor/ligand percent).

Further pathway analysis revealed distinct signaling adaptations across T cell subsets and tumor models. Activated Cd8^+^ T cells from LLC1 tumors showed enrichment in pathways related to T cell receptor signaling, spliceosome activity, pyrimidine metabolism, HIF1 and TNF signaling, consistent with increased activation, proliferation, and inflammatory signaling. Kras^LA2^-activated Cd8^+^ T cells favored mTOR and AMPK signaling, choline metabolism, and platinum drug resistance, highlighting metabolic stress adaptation and effector reprogramming. Cd4^+^ T cells in LLC1 were metabolically active, with enrichment in oxidative phosphorylation, HIF1, MAPK, mTOR, and NF−κB pathways. In contrast, Kras^LA2^ Cd4^+^ cells engaged NK cell-mediated cytotoxicity, Fc-gamma receptor-mediated phagocytosis, phosphatidylinositol signaling, and platelet activation, indicating enhanced innate immune crosstalk and microenvironment remodeling. Cd8^+^ memory T cells in LLC1 activated TNF, FOXO, and pyrimidine metabolism pathways, supporting the immune signaling and metabolic demands of memory maintenance. Kras^LA2^ memory T cells enriched peroxisome, glutathione metabolism, Notch signaling, and inositol phosphate metabolism, reflecting oxidative stress responses and differentiation. Bulk Cd8^+^ T cells in LLC1 showed enrichment of MAPK signaling, mRNA surveillance, transcriptional misregulation in cancer, and lysine degradation, consistent with active signal transduction and transcriptional control. Kras^LA2^ Cd8^+^ cells favored proteasome activity, FCER1 signaling, amino sugar and nucleotide sugar metabolism, and N-glycan biosynthesis, suggesting elevated protein turnover, immune receptor activity, and glycosylation. Th17 cells in LLC1 were enriched in proteasome function, antigen presentation, and TNF signaling, reflecting strong inflammatory activity, while Kras^LA2^ Th17 cells enriched inositol phosphate metabolism, lysine degradation, and branched-chain amino acid catabolism, highlighting metabolic rewiring. Tregs in LLC1 displayed MAPK, NF−κB, and proteasome pathway activation, consistent with inflammatory tolerance and protein degradation. Kras^LA2^ Tregs enriched NK cell-mediated cytotoxicity, Notch signaling, FCER1 signaling, and glycerophospholipid metabolism, indicating roles in innate immune modulation and lipid metabolism (**Figure 6A**).

Moreover, receptor-ligand analysis revealed distinct T cell communication networks across models. In the Kras^LA2^ model, Tregs prominently expressed the *Cd3d* receptor, which bound H2-K1 ligands on activated Cd8^+^ T cells. Additional interactions involved the *Cd3g* receptor on Tregs recognizing *B2m* and *H2-K1* on activated Cd8^+^ T cells, indicating tight regulatory-effector crosstalk. Activated Cd8^+^ T cells also exhibited *Ptprc* receptors interacting with *Lgals1* ligands on other activated Cd8^+^ T cells, suggesting intra-subset signaling. The LLC1 TME displayed a broader interaction landscape. The *Cd3d* receptor, expressed across multiple T cell subsets, including activated Cd8^+^, Cd4^+^, Cd8^+^ memory T cells, and Tregs, engaged *H2-K1* ligands on activated Cd8^+^ T cells. Weaker interactions included *Cd2* receptors on Tregs binding *Cd48* ligands on Cd8^+^ T cells, and *Cd69* on Cd8^+^ memory T cells interacting with *Lgals1* on activated Cd8^+^ cells, potentially reflecting activation or exhaustion signaling. In healthy controls, interactions were more limited and focused primarily on *Cd3d* receptors on Cd4^+^ and Cd8^+^ T cells binding *H2-K1* ligands on activated Cd8^+^ T cells. Additionally, activated Cd8^+^ T cells engaged in homotypic signaling through *Thy* receptors interacting with *Itgb2* ligands within the same subset, consistent with homeostatic immune surveillance (**Figures 6B, C**). This integrated view highlights how tumor microenvironments shape T cell composition, functional programming, and intercellular communication, with LLC1 tumors promoting inflammatory activation and broad T cell crosstalk, Kras^LA2^ tumors driving metabolic and stress adaptations with focused regulatory interactions, and healthy tissue maintaining homeostatic signaling and surveillance.

### 3.4 Tumor-driven remodeling and functional specialization of NK cell subsets in lung cancer models

**Figure 7:**
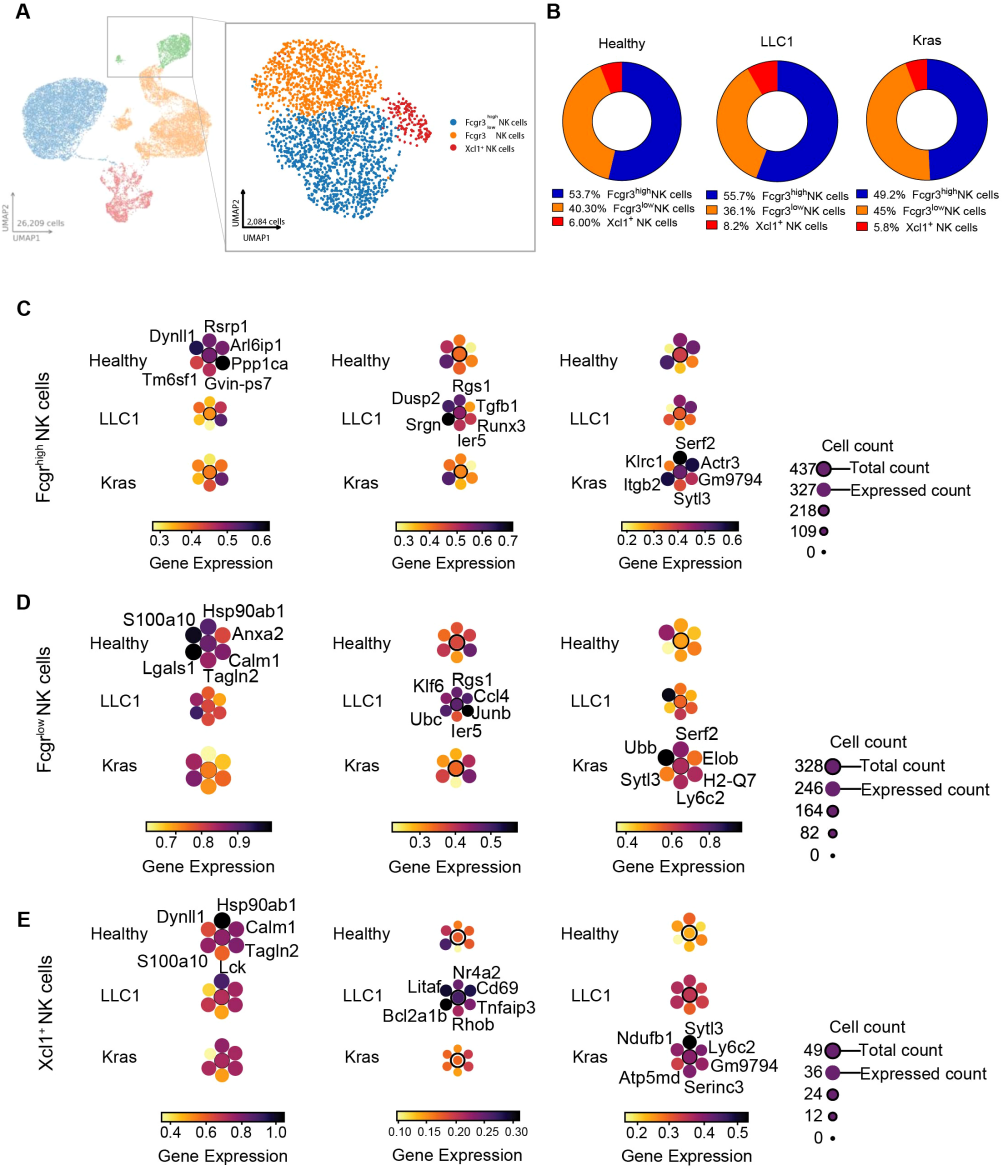
Tumor-driven remodeling and functional specialization of NK cell subset in lung cancer models. **(A)** UMAP plots illustrate major immune cell compartments identified among CD45^+^ cells (left), and refined subcluster annotations within the NK cell compartment (right), including Fcgr3^high^ NK cells, Fcgr3^low^NK cell and Xcl1^+^ NK cells. **(B)** Pie chart representing the percentage of NK cell subclusters within the CD45^+^ cell population for all three models (healthy, LLC1 and Kras^LA2^). **(C–E)** Planet plots of Fcgr3^high^ NK cells **(C)**, Fcgr3^low^ NK cells **(D)**, Xcl1^+^ NK cells **(E)** illustrating 6 distinct marker genes in healthy, LLC1 and Kras^LA2^ mouse models, featuring a central circle representing aggregated gene expression and surrounding circles depicting individual gene expression levels.

NK cells, representing the innate lymphocyte compartment, were identified among the major CD45^+^ immune clusters. Subclustering revealed three transcriptionally distinct NK cell subsets based on canonical marker gene expression: Fcgr3^high^ NK cells, Fcgr3^low^ NK cells, and Xcl1^+^ NK cells (**Figures 7A**; **Supplementary Figure S6A**). The relative proportions of these subsets varied across models. The Fcgr3^high^ NK cell population was reduced in Kras^LA2^ tumors (49.2%) compared to healthy lungs (53.7%) and LLC1 tumors (55.7%). In contrast, Fcgr3^low^ NK cells were increased in Kras^LA2^ tumors (45%) but reduced in LLC1 tumors (36.1%) relative to healthy lungs (40.3%). The Xcl1^+^ NK cell population was slightly expanded in LLC1 tumors (6%) compared to Kras^LA2^ tumors (5.8%) and healthy lungs (6%) (**Figure 7B**), while proportion analysis was not significantly changed (**Supplementary Figure S6B**). However, the observed relative proportional shifts suggest tumor-specific remodeling of NK cell subset composition, with Kras^LA2^ tumors favoring Fcgr3^low^ NK cells and LLC1 tumors promoting Xcl1^+^ NK cells. Transcriptional programs of NK cell subsets revealed distinct model-dependent functional adaptations. Fcgr3^high^ NK cells in Kras^LA2^ tumors upregulated genes related to NK cell activation, migration, and cytoskeletal remodeling (*Klrc1, Itgb2, Actr3*), consistent with an activated but regulated phenotype. In LLC1 tumors, Fcgr3^high^ NK cells expressed genes associated with immune suppression and altered signaling (*Tgfb1, Rgs1, Dusp2*), indicating a more suppressive microenvironment. Healthy Fcgr3^high^ NK cells maintained homeostatic gene expression patterns (**Figures 7C**; **Supplementary Figure S6C**). Fcgr3^low^ NK cells exhibited divergent functional programs. Kras^LA2^ tumors upregulated genes involved in immune activation and antigen presentation (*Ly6c2, H2-Q7*), consistent with an activated NK cell phenotype. In contrast, LLC1 tumors expressed genes associated with immune signaling and inflammation (*Ccl4, Junb*), suggesting a more inflammatory or dysregulated environment. Healthy Fcgr3^low^ NK cells showed homeostatic and cytoskeletal organization programs (**Figures 7D**; **Supplementary Figure S6D**). Xcl1^+^ NK cells demonstrated model-specific metabolic and immune adaptations. Kras^LA2^ tumors upregulated genes linked to mitochondrial activity and immune engagement (*Ndufb1, Ly6c2*), reflecting a metabolically active phenotype. LLC1 tumors increased expression of inflammation- and activation-related genes (*Cd69, Tnfaip3*), indicative of immune regulation and resistance to apoptosis. Healthy Xcl1^+^ NK cells expressed homeostatic and cytoskeletal organization genes (**Figures 7E**; **Supplementary Figure S6E**).

**Figure 8:**
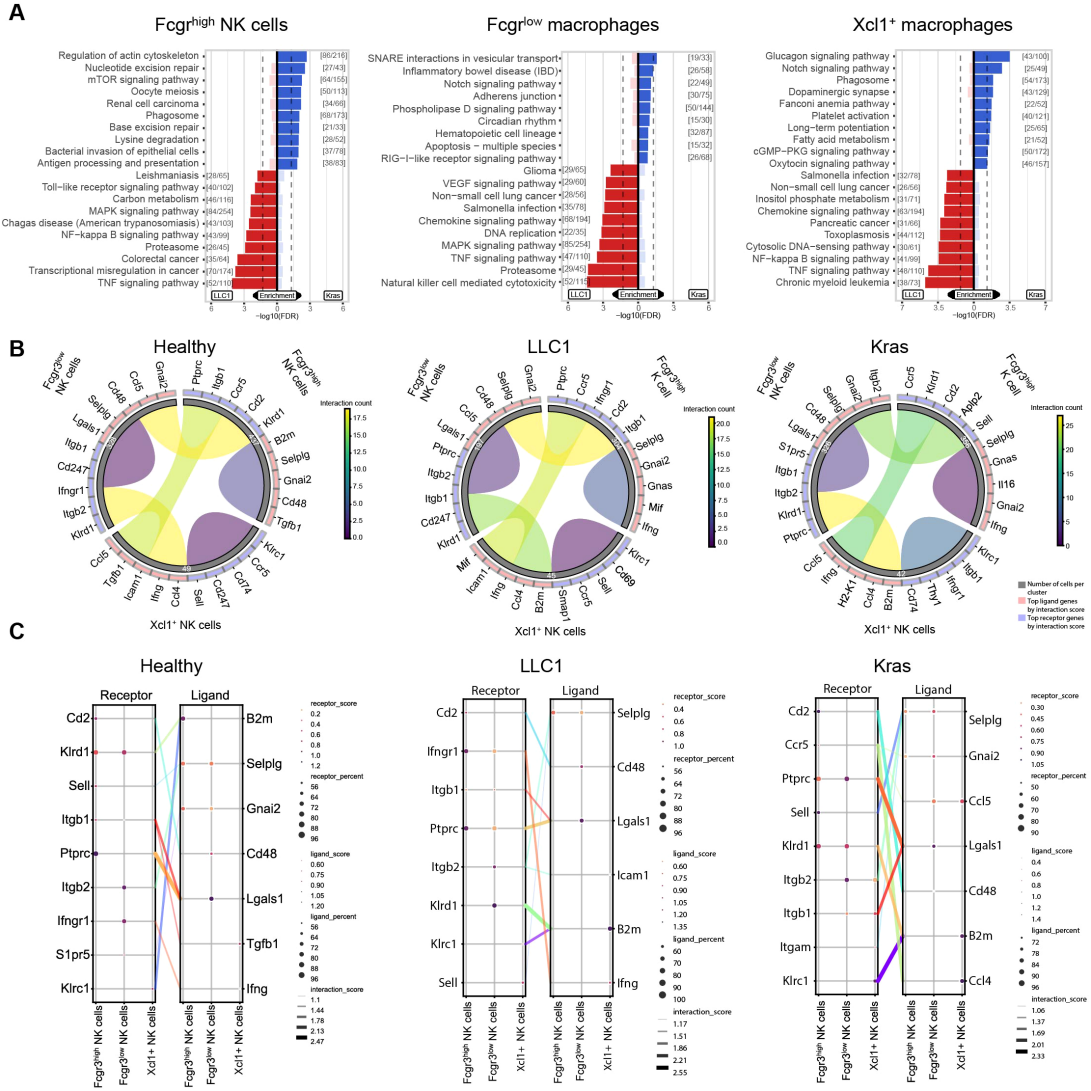
Kras^LA2^ and LLC1 tumors drive divergent NK cell states, ranging from metabolic and regulatory to inflammatory and suppressive phenotypes. **(A)** Pathway enrichment analysis using the KEGG database highlights upregulated pathways in LLC1 compared to Kras^LA2^ mouse models for each cell subcluster (Fcgr3^high^ NK cells, Fcgr3^low^ NK cells, and Xcl1^+^ NK cells). **(B)** Cyclone plots show the overall communication patterns between different cell subclusters, emphasizing the top five receptors and ligands for each cluster. **(C)** Connection plots illustrate the most significant ligand-receptor interactions between clusters across lung cancer models (healthy, LLC1, and Kras^LA2^) by displaying distinct receptor-ligand pairs that are enriched (receptor/ligand score) with a certain cell coverage (receptor/ligand percent).

Pathway analysis further highlighted tumor-specific NK cell programs. Fcgr3^high^ NK cells in Kras^LA2^ tumors showed enrichment of pathways related to actin cytoskeleton regulation, mTOR signaling, phagosome, and antigen processing and presentation, consistent with a metabolically active state and enhanced antigen handling. In LLC1 tumors, Fcgr3^high^ NK cells were enriched for TNF, NF−κB, MAPK, Toll-like receptor, and transcriptional misregulation pathways, indicating a pro-inflammatory, activated phenotype. Fcgr3^low^ NK cells in Kras^LA2^ tumors were enriched for Notch, phospholipase D, and RIG-I-like receptor signaling, reflecting regulatory and immune-sensing functions, while LLC1 Fcgr3^low^ NK cells showed enrichment of NK cell-mediated cytotoxicity, proteasome, TNF, MAPK, and VEGF signaling pathways, consistent with an activated cytotoxic and inflammatory state (**Figure 8A**). Receptor-ligand analyses revealed both conserved and model-specific interactions. Fundamental *Klrc1-B2m* and *Ptprc-Lgals1* interactions were maintained across all models. In Kras^LA2^ tumors, *Xcl1^+^* NK cells interacted with Fcgr3^high^ and Fcgr3^low^ NK cells through *Itgb1/Lgals1* and *Itgb2/Selplg*, respectively, while Fcgr3^high^ and Fcgr3^low^ NK cells engaged in *Klrd1-B2m* and *Ptprc-Lgals1* interactions. LLC1 tumors showed similar *Klrc1-B2m* and *Klrd1-B2m* interactions, as well as a unique *Cd2-Cd48* interaction between Fcgr3^high^ and Fcgr3^low^ NK cells. In healthy lungs, interactions were limited to homeostatic *Klrc1-B2m* and *Ptprc-Lgals1* pairs. These results suggest that Kras^LA2^ and LLC1 tumors induce additional migration- and regulation-related NK cell interactions, reflecting tumor-specific functional adaptations (**Figures 8B, C**). Overall, these data indicate that NK cells undergo tumor-specific compositional and functional remodeling, with Kras^LA2^ tumors promoting metabolically active, regulatory Fcgr3^low^ NK cells and LLC1 tumors favoring inflammatory or suppressive NK cell phenotypes, while healthy lungs maintain homeostatic programs.

### 3.5 Activation and immunometabolic rewiring define distinct myeloid landscapes in LLC1 and Kras^LA2^ tumors

**Figure 9:**
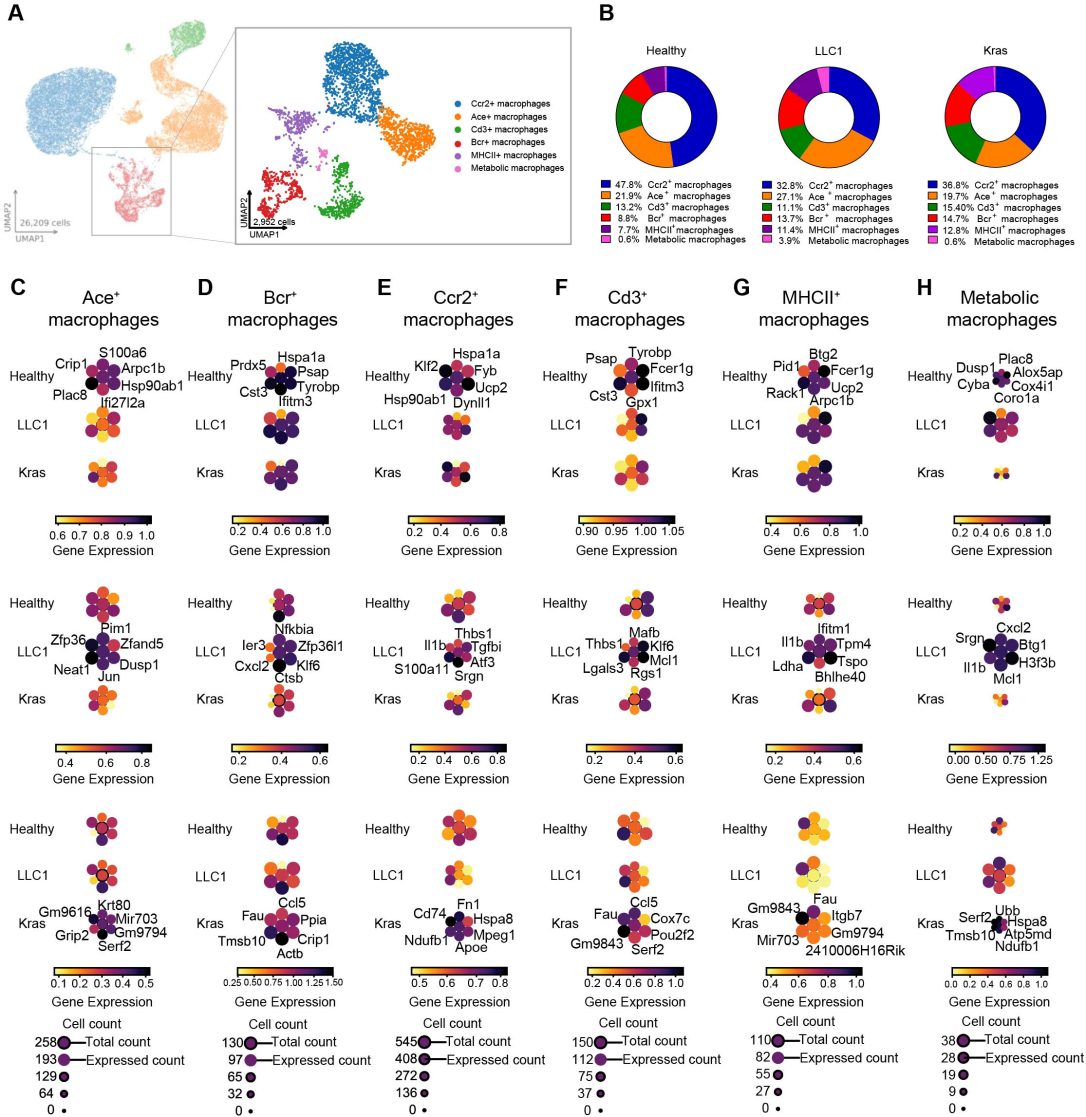
Activation and immuno-metabolic rewiring define distinct myeloid landscapes in LLC1 and Kras tumors. **(A)** UMAP plots show major immune cell compartments identified among CD45^+^ cells (left) and refined subcluster annotations within the macrophage compartment (right), including Ace^+^ macrophages, Bcr^+^ macrophages, Ccr2^+^ macrophages, Cd3^+^ macrophages, metabolic macrophages, and MHCII^+^ macrophages. **(B)** Pie chart showing the percentage of macrophage subclusters within the CD45^+^ cell population for all three models (healthy, LLC1, and Kras^LA2^). **(C–H)** Planet plots of Ace^+^ macrophages **(C)**, Bcr^+^ macrophages **(D),** Ccr2^+^ macrophages **(E)**, Cd3^+^ macrophages **(F)**, MHCII^+^ macrophages **(G)**, and metabolic macrophages **(H)** illustrating six distinct marker genes in healthy, LLC1, and Kras^LA2^ mouse models, with a central circle representing aggregated gene expression and surrounding circles showing individual gene expression levels.

To further analyze the immune cell composition within the tumor microenvironment, we next focused on the macrophage compartment. Canonical marker-based subclustering analysis identified six transcriptionally distinct macrophage subsets: Ace^+^ macrophages, Bcr^+^ macrophages, Ccr2^+^ macrophages, Cd3^+^ macrophages, metabolically active macrophages, and MHCII^+^ macrophages. To exclude T cell-macrophage doublets, we performed flow cytometry analysis (**Supplementary Figures S7A, B**) and immunofluorescence staining (**Supplementary Figure S7C**), which confirmed co-expression of macrophage and T cell markers within single cells in Kras lung tumor. Furthermore, the distribution of these subpopulations differed markedly among the Kras, LLC1, and healthy control models (**Figures 9A**, **Supplementary Figure S8A**). Ccr2^+^ macrophages were most abundant in healthy controls (47.8%) and remained a major subset in Kras^LA2^ (36.8%) and LLC1 (32.8%) lungs. Ace^+^ macrophages represented a larger fraction in LLC1 (27.0%) compared to healthy (21.3%) and Kras^LA2^ (19.7%) models. Cd3^+^ macrophages were present at moderate levels in Kras^LA2^ (15.4%), healthy (13.2%), and LLC1 (11.1%) groups. Bcr^+^ macrophages showed slightly higher representation in Kras^LA2^ (14.7%) and LLC1 (13.6%) relative to healthy tissue (8.8%). MHCII^+^ macrophages were less frequent but detectable across all models (Kras^LA2^: 12.8%, LLC1: 11.4%, healthy: 7.4%). Metabolic macrophages were rare overall, but reached a relative peak in LLC1 (3.98%) and remained low in Kras^LA2^ (0.6%) and healthy (0.6%) lungs (**Figure 9B**). Although these proportional shifts are not significant (**Supplementary Figure S8B**), the compartment’s transcriptional landscape changed. Dominant clusters such as Ccr2^+^ macrophages in healthy tissue drove homeostatic and stress-response signatures (*Hspa1a, Klf2, Hsp90ab1*), while in tumor contexts (Kras^LA2^ and LLC1), these cells increasingly acquired pro-inflammatory, profibrotic, and matrix-remodeling programs characterized by upregulation of *Fn1, Cd74, Apoe, Hspa8, Ndufb1*, and *Mpeg1* in Kras^LA2^, and *Il1b, Thbs1, S100a11*, and *Tgfbi* in LLC1, reflecting adaptation to the TME (**Figures 9E**, **Supplementary Figure S9C**). Ace^+^ macrophages in the Kras^LA2^ model upregulated genes linked to EMT and tumor invasion (*Krt80*), suggesting a pro-metastatic phenotype, while LLC1 Ace^+^ macrophages favored immune suppression and inflammation regulation (*Pim1, Zfp36*). In healthy lungs, Ace^+^ cells expressed homeostatic markers (*S100a6, Plac8*) (**Figures 9C**; **Supplementary Figure S9A**). Bcr^+^ macrophages in Kras^LA2^ upregulated chemotactic and cytoskeletal genes (*Ccl5, Fau, Tmsb10, Actb*), indicating support for immune recruitment and tissue remodeling, whereas in LLC1, these cells exhibited a signature of inflammatory regulation (*Nfkbia, Ier3, Cxcl2*), and in healthy tissue, stress response and immune surveillance genes (*Hspa1a, Prdx5, Tyrobp*) (**Figures 9D**; **Supplementary Figure S9B**). For Cd3^+^ macrophages, Kras^LA2^ tumors induced *Ccl5, Fau, Pou2f2*, and *Cox7c* (recruitment, cytoskeletal, metabolic adaptation), while LLC1 promoted *Mafb, Thbs1, Lgals3*, and *Mcl1* (differentiation, ECM interaction, survival), and healthy tissues favored immune homeostasis (*Tyrobp, Psap, Ifitm3*) (**Figures 9F**; **Supplementary Figure S9D**). Metabolic macrophages revealed model-dependent profiles: Kras^LA2^ samples were marked by oxidative phosphorylation and protein turnover (*Ubb, Serf2, Tmsb10, Ndufb1, Atp5md, Hspa8)*, LLC1 by pro-inflammatory and survival genes (*Cxcl2, Il1b, Srgn, Mcl1, H3f3b*), and healthy by metabolic regulation (*Plac8, Dusp1, Cyba, Cox4i1*) (**Figures 9H**; **Supplementary Figure S9E**). MHCII^+^ macrophages in Kras^LA2^ expressed *Fau*, *Mir703*, and *Itgb7*, indicating possible tumor-specific immune roles; LLC1 upregulated inflammatory and metabolic programs (*Il1b, Ifitm1, Ldha, Bhlhe40*), while healthy MHCII^+^ cells maintained immune surveillance-associated signatures (*Btg2, Rack1, Ucp2, Fcer1g*) (**Figures 9G**; **Supplementary Figure S9F**).

**Figure 10:**
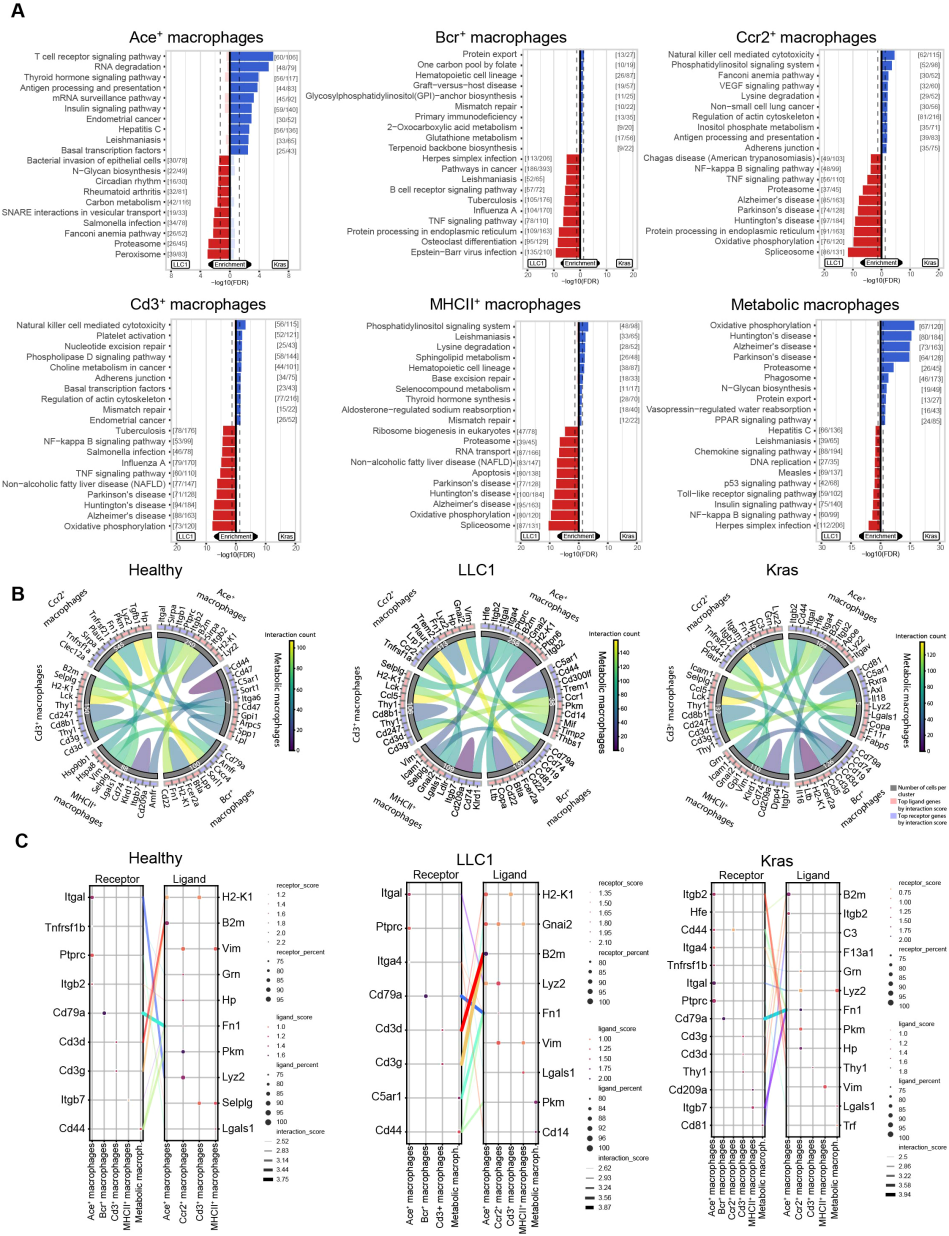
Divergent macrophage communication landscapes define inflammatory versus pro-tumorigenic remodeling in LLC1 and Kras^LA2^ lung tumors. **(A)** Pathway enrichment analysis using the KEGG database highlights the upregulated pathways in LLC1 versus Kras^LA2^ mouse models for each cell subcluster (Ace^+^ macrophages, Bcr^+^ macrophages, Ccr2^+^ macrophages, Cd3^+^ macrophages, metabolic macrophages, and MHCII^+^ macrophages). **(B)** Cyclone plots depict the overall communication patterns between different cell subclusters. The top five receptors and ligands for each cluster are emphasized in the visualization. **(C)** Connection plots illustrate the most significant ligand-receptor interactions between clusters across lung cancer models (healthy, LLC1 and Kras^LA2^) by showing distinct receptor-ligand pairs that are enriched (receptor/ligand score) with a certain cell coverage (receptor/ligand percent).

Pathway analysis reinforced these patterns, revealing that the global transcriptional frame of the macrophage compartment was molded by both the dominance of specific subsets and their model-specific state. For example, Kras^LA2^ macrophages were enriched for NK cell-mediated cytotoxicity, antigen presentation, VEGF, and lipid metabolism pathways, while LLC1 showed upregulation of NF−κB, TNF, and oxidative phosphorylation pathways, driven mostly by Ace^+^, Ccr2^+^, and metabolic macrophage clusters (**Figure 10A**). Kras^LA2^ tumors exhibited targeted ligand-receptor crosstalk among Ace^+^, Bcr^+^, and MHCII^+^ subsets, whereas LLC1 displayed broader inter-subcluster interactions. Overall, shifts in macrophage composition aligned with model-specific pathway activation and communication patterns, linking dominant subsets to adaptations in immune regulation, metabolism, and intercellular signaling. Receptor-ligand analysis further reflected differences in macrophage communication across conditions. In Kras^LA2^ tumors, MHCII^+^ (*Itgb7-Fn1*) and Bcr^+^ (*Cd79a*-*Fn1*) macrophages formed focused interactions with Ccr2^+^ subsets, while Ace^+^ cells interacted with Ccr2^+^ and metabolic macrophages via *Itgal-Lyz2* and *Itgb2-Hp*. LLC1 tumors showed broader connectivity, including *Cd79a-Fn1* between Bcr^+^ and Ccr2^+^, *Itgal-Lyz2* among Ace^+^, Ccr2^+^, and Ace^+^ cells, *Cd3d/Cd3g-B2m* between Cd3^+^ and Ace^+^, and *Cd44-Pkm* within the metabolic cluster. Healthy lungs exhibited limited, conserved interactions centered on Bcr^+^, metabolic, and Ccr2^+^ macrophages (**Figures 10B, C**). Thus, LLC1 featured the richest macrophage network, Kras^LA2^ favored targeted links, and healthy tissue maintained homeostatic signaling. Macrophage compartments in healthy, LLC1, and Kras^LA2^ models display marked shifts in subset abundance, transcriptional programming, pathway activation, and intercellular communication; LLC1 tumors are characterized by extensive cross-subcluster interactions and metabolic/inflammatory adaptation, while Kras^LA2^ tumors exhibit focused, pro-tumorigenic signaling and healthy tissue maintains homeostatic communication.

## 4 Discussion

This study used scRNA-seq to map the immune landscape in healthy, LLC1, and Kras^LA2^-driven lung cancer models, revealing significant model-dependent remodeling of the immune microenvironment that affected the abundance, transcriptional states, pathway activity, and intercellular interactions of all major immune cell compartments. The LLC1 model showed marked expansion of B cells and reduced proportions of T cells and NK cells, indicating adaptive immune suppression along with inflammatory and stress-related transcriptional programs. In contrast, the Kras^LA2^ model maintained a balanced immune composition but induced distinct functional remodeling, including unique transcriptional programs and novel cell-to-cell communication networks. Healthy lungs exhibited a stable mix of B cells, T cells, NK cells, and macrophages, with well-preserved homeostatic programs. Distinct immune subpopulations, including B cells, T cells, NK cells, and macrophages, displayed context-specific gene expression and pathway enrichment, revealing specialized immune activation, metabolic reprogramming, and cytotoxic responses depending on the tumor model.

The dramatic B cell expansion observed in LLC1 tumors (62.7%) compared to healthy lungs (36.8%) aligns with recent findings that B cell infiltration and tertiary lymphoid structure formation are critical determinants of immunotherapy response in lung cancer. One study demonstrated that tumor-infiltrating B cells have dual roles, with regulatory B cell subsets secreting immunosuppressive cytokines such as IL-10 and TGF-β, which promote tumor progression (22). Our findings of stress-related transcriptional programs in LLC1 B cells (upregulation of *Ier5, Jun, Fos*) support this immunosuppressive phenotype, consistent with recent evidence that B cell heterogeneity within the TME determines therapeutic outcomes (23, 24). In contrast, the Kras^LA2^ model’s preservation of a balanced immune composition with mature B cell dominance (81.5% of the B cell compartment) reflects a different adaptive strategy. The upregulation of pro-inflammatory genes (*Ccl5, Nfkbid, Eno1*) in Kras^LA2^ mature B cells suggests active immune engagement rather than suppression. This pattern aligns with recent work showing that functionally active B cells within tertiary lymphoid structures contribute to anti-tumor immunity through antibody production and T cell activation (24). The specific upregulation of *Ccl5* in Kras^LA2^ mature B cells is particularly significant, as *Ccl5* has emerged as a critical orchestrator of immune cell recruitment and activation in the tumor microenvironment. It was demonstrated that *Ccl5* production by Kras-mutant lung cancer cells creates complex immunomodulatory effects, with the chemokine serving dual roles in both immune activation and suppression depending on the cellular context (25). The same study showed that Kras^G12C^ mutations drive Ccl5 production through MAPK/ERK signaling, creating a recruitment signal for both effector and regulatory immune cell populations (25).

Furthermore, the context-specific T cell dysfunction observed across models provides important insights into immune escape mechanisms. In the LLC1 model, the upregulation of anti-apoptotic mechanisms and impaired migration markers (*Mcl1, Dusp2, Klf6, Rgs1*) in activated Cd8^+^ T cells reflects the immunosuppressive microenvironment characteristic of this model. These findings are consistent with recent single-cell analyses, which identified exhausted Cd8^+^ T cells expressing high levels of *Pdcd1* and *Lag3* while maintaining proliferative capacity and cytokine production (26). In contrast, the signatures in Kras-associated Cd8^+^ T cells (*Zeb2, Pdcd4, Ly6c2*) suggest a more robust but potentially exhausted response. This pattern aligns with findings from a study demonstrating that tumor-associated Cd8^+^ T cells express both exhaustion markers and effector molecules, indicating functional heterogeneity within the exhausted compartment (27). The enrichment of metabolic reprogramming pathways (mTOR, AMPK signaling) in Kras^LA2^ T cells further supports evidence that metabolic adaptation is crucial for T cell persistence in the TME (28, 29). Additionally, the model-specific NK cell adaptations revealed in our analysis align with the emerging understanding of NK cell plasticity in cancer. The functional suppression observed in Kras NK cells, characterized by expression of *Klrc1*, *Psme2b*, and *Ly6c2*, is consistent with recent work showing rapid functional impairment of NK cells following tumor entry. Analysis of human colorectal tumors identified similar patterns of cytotoxicity downregulation in tumor-resident NK cells compared to newly infiltrating populations (30). The inflammatory and stress-related transcriptional programs in LLC1 NK cells (*Fos, Dusp2, Klf6*) reflect the complex microenvironmental pressures that reshape innate immune function. NK cell exhaustion in lung cancer correlates with immunosuppressive cytokine exposure and metabolic dysfunction (31), supporting our pathway enrichment findings showing peroxisome and phospholipase D signaling activation in LLC1 NK cells.

Moreover, the heterogeneity of macrophages observed across our models reflects the well-documented plasticity of tumor-associated macrophages (TAMs) in lung cancer. Recent literature and meta-analyses emphasize that TAMs exist along a complex spectrum of activation states rather than conforming to the simple M1/M2 classification system. M1-like macrophages typically provide tumor-suppressive functions through inflammatory cytokine production. M2 macrophages, in contrast, can be further classified into four subtypes, M2a, M2b, M2c, and M2d, based on the stimuli that activate them. M2a and M2b are primarily associated with immunomodulation and the promotion of Th2-related responses, while M2c and M2d are mainly involved in immunosuppression and tissue remodeling (32). Single-cell transcriptomic profiling in NSCLC has revealed an unexpectedly high degree of macrophage heterogeneity, identifying not only alveolar and tumor-associated populations but also marked differences in gene expression and metabolic adaptation between macrophage subtypes within tumors and adjacent lung tissue (33). TAMs undergo dynamic metabolic reprogramming, shifting between glycolysis and oxidative phosphorylation in response to local cytokine cues such as IFNG and IL4, as well as tumor-derived signals. These metabolic states are directly linked to specific functional programs, including immunosuppression and promotion of tissue fibrosis (34). Our findings directly support and extend this paradigm: the model-dependent transcriptional signatures observed in our Ace^+^, Bcr^+^, Ccr2^+^, Cd3^+^, metabolic, and MHCII^+^ macrophage subsets demonstrate this type of context-specific adaptation. For example, the enrichment of oxidative phosphorylation, NF−κB, and TNF signaling pathways in LLC1 macrophages, versus the cytotoxic, antigen presentation, and metabolic pathway signatures in Kras macrophages, illustrates how tumor genetics can fundamentally reshape macrophage functional programs. Among the diverse macrophage subpopulations identified in this lung cancer study, Cd3^+^ macrophages may represent a particularly intriguing and understudied subset that exhibits profound model-dependent functional adaptations within the TME as shown in human lung cancer TME by Kaminskis group, where macrophages express TCR repertoires (35). To address the possibility that the Cd3^+^ macrophage cluster identified by scRNA-seq may represent T cell/macrophage doublets or tightly interacting cells-artifacts known to occur in droplet-based single-cell platforms and difficult to fully exclude computationally-we performed complementary validation experiments. Using flow cytometry and immunofluorescence staining, we observed cells co-expressing macrophage and T cell markers within mouse lung tumors. While these data support the presence of dual-marker positive cells, we acknowledge that technical and biological complexities remain. Notably, previous studies in infectious and inflammatory settings have reported CD3^+^ macrophages or CD3/TCR-expressing myeloid cells, suggesting that such populations may exist under specific conditions (36, 37). In Kras-driven tumors, these cells upregulate genes such as *Ccl5, Fau, Pou2f2*, and *Cox7c*, indicating roles in chemokine-mediated recruitment, cytoskeletal remodeling, and metabolic adaptation within the tumor microenvironment. Their pathway enrichments for NK cell-mediated cytotoxicity, platelet activation, and phospholipase D signaling support a context-specific cytotoxic or immunomodulatory function. In contrast, in LLC1 tumors, Cd3^+^ macrophages express *Mafb, Thbs1, Lgals3*, and *Mcl1*, with dominant signatures of oxidative phosphorylation, TNF, and NF−κB signaling, suggesting a more inflammatory and pro-tumoral phenotype. Notably, receptor-ligand analysis revealed broader interaction networks in LLC1, including unique *Cd3d* and *Cd3g* contacts with *B2m* Ace^+^ macrophages, implicating Cd3^+^ macrophages in crosstalk with other myeloid populations and potentially regulatory circuits. These findings align with emerging literature describing CD3^+^ macrophages as a unique and functionally plastic subset in tumor and inflammatory settings. The presence of a CD3^+^ macrophage subpopulation in hepatocellular carcinoma have been associated with improved patient survival (36). Phenotypic characterization has further shown that CD3^+^ macrophages, derived from circulating human monocytes, can exhibit both TCRαβ^+^ and TCRαβ^−^ profiles and are capable of secreting pro-inflammatory cytokines in response to CD3 and TNF mediated pathways (37). However, the functional consequences of CD3 expression on macrophages remain incompletely defined, with ongoing debate as to whether they predominantly drive anti-tumor inflammation or, depending on the context, facilitate immune regulation and tissue repair. To our knowledge, there are currently no published reports documenting the presence of CD3^+^ macrophages in human lung cancer tissue. The distinct pathway enrichments and receptor-ligand interaction networks identified in each tumor model have important therapeutic implications. In LLC1 tumors, extensive intercellular communication, including novel Cd3^+^ macrophage interactions and broad T cell receptor-ligand networks, suggests that overcoming the immunosuppressive microenvironment may require combination immunotherapies targeting multiple immune cell types. In contrast, Kras tumors are characterized by enrichment of metabolic reprogramming pathways such as glutathione metabolism, N-glycan biosynthesis, and proteasome activity, which represent promising targets to restore immune function. This is consistent with emerging clinical evidence supporting metabolic pathway inhibition in Kras-mutant lung cancers (38).

Generally, Kras^LA2^ model in our study is characterized by metabolic vulnerability and adaptation, with enrichment of mTOR, AMPK signaling and glutathione metabolism. Recent literature showed that Kras signaling drives extensive reprogramming of lipid metabolism within the TME, leading to significant accumulation of intracellular lipid metabolites. This lipid burden promotes immunosuppression through multiple mechanisms. It directly impairs T cell-mediated cytotoxicity and enhances the pro-tumorigenic function of Cd4^+^FoxP3^+^Treg cells (39). In addition, Kras mutations increase glycolysis metabolism and hypoxia pathway activation, which restricts CD8^+^PD-1 T infiltration and suppresses the anti-tumor immune response. The high expression of *CIB1*, induced by Kras, regulates glycolytic metabolism, further restricting CD8^+^PD-1 T infiltration and suppressing immune responses (39). This could, for example, validate the use of the Kras^LA2^ model to study strategies, such as metabolic inhibition, aimed at restoring T cell fitness.

Conversely, the LLC1 model is unified by a widespread, pro-tumoral inflammatory signature dominated by the NF−κB and TNF signaling pathways found across its B cells, T cells, NK cells, and macrophages. This persistent activation of NF−κB/TNF is consistent with studies showing that myeloid-derived NF−κB activity drives a protumor phenotype in lung cancer, resulting in chronic inflammation that fails to clear the tumor (40). In addition, NF−κB in the tumor microenvironment promotes an immunosuppressive immune cell state, activates fibroblasts to remodel the extracellular matrix, and increases vascular permeability through endothelial cell modulation. It can also reprogram tumor metabolism by enhancing glycolysis and lipid synthesis while suppressing oxidative phosphorylation, facilitating metabolic flexibility (41). Importantly, while NF−κB has been implicated in promoting tumor growth via inflammation and immune evasion, its role is multifaceted; certain studies show NF−κB activity correlates with enhanced T cell infiltration and anti-tumor immunity, indicating a complex balance depending on cellular context and tumor genotype. These results confirm that effective immunotherapy strategies must be stratified by genotype, targeting metabolic compromise in Kras-driven tumors and focusing on disrupting the central inflammatory and survival axes (NF−κB/TNF) in the LLC1-like microenvironment.

Understanding the similarities and differences between human and mouse lung cancer cell populations is crucial for advancing translational research and developing effective therapies. Zilionis et al. developed a cross-species, single-cell transcriptomic atlas of the tumor-infiltrating myeloid cell (TIM) landscape. This atlas revealed conserved and divergent patterns that have significant implications for immunotherapy (42). The researchers demonstrated significant conservation, particularly among dendritic cells, monocytes, and specific neutrophil phenotypes. Additionally, they observed a broad conservation of major gene expression programs between human and mouse immune cells. The study found that cell type identity, rather than the source organism, dictates similarity in gene expression profiles. However, species-specific patterns in macrophages highlight the importance of being cautious when translating findings from mouse models to human therapies (42). While the comparative analysis establishes foundational cross-species conservation in basic myeloid structure, our single-cell data demonstrate that tumor genetics reshape the immune microenvironment’s functional state and therapeutic vulnerability. Specifically, the LLC1 model fosters an immunosuppressive landscape characterized by B cell expansion, inflammatory dysfunction in NK cells, and anti-apoptotic signatures in Cd8^+^ T cells. This suggests that overcoming resistance will necessitate combination therapies targeting multiple regulatory cells. In stark contrast, the Kras^LA2^ model maintains a balanced immune composition but drives distinct functional remodeling. Notably, it enriches metabolic reprogramming pathways in T cells and has model-specific functions of the novel Cd3^+^ macrophage population.

Beyond shared myeloid features, LLC1 and Kras model distinct lung adenocarcinoma states that mirror major genomic subclasses of human NSCLC. LLC1 is hypermutated (>20,000 somatic mutations), whereas Kras tumors harbor few coding single-nucleotide variants (SNVs) but extensive copy number alterations and Kras amplifications (43, 44), reflecting the two dominant genomic architectures of human tumors (44). Kras evolves through chromosomal instability, while LLC1 accumulates clustered mutations driven by multiple DNA damage and repair processes (43, 44). Histologically, both align with lung adenocarcinoma but capture distinct morphologies: Kras^LA2^ resembles Kras-mutant solid lung adenocarcinoma, whereas LLC1 reflects bronchioloalveolar, type II pneumocyte-derived adenocarcinoma (43, 44). Together, these models represent complementary human lung adenocarcinoma type. Consistent with this framework, tumor genotype dictates immune organization: LLC1 promotes an NF-κB/TNF-driven immunosuppressive milieu, whereas Kras-driven tumors exhibit metabolic exhaustion and adaptive immune remodeling, highlighting genotype-specific therapeutic vulnerabilities in NSCLC.

It is important to acknowledge several limitations of this study. Pooling samples masks inter-animal biological variability, limiting statistical inference unless donor identity is preserved through barcoding or genotype-based demultiplexing. Multiplexing approaches that retain donor information can restore the ability to detect inter-individual effects and enable population-level analyses, such as donor-specific differential expression (45, 46). While optimized pooling strategies that balance pool size, number of pools, and sequencing depth can remain cost-effective and statistically informative, they require careful experimental design. In settings with high within-group transcriptional variability, small RNA sample pools may reduce noise and partially compensate for reduced biological replicates; however, this comes at the expense of detecting individual-level differences (47). Accordingly, our data provide a descriptive overview of the aggregate immune landscape in each lung cancer model rather than a robust assessment of population-level variability, underscoring the need for future studies incorporating individual mouse replicates for validation.

## 5 Conclusions

This study provides a comprehensive single-cell map of CD45^+^ immune cells in healthy, LLC1, and Kras^LA2^-driven lung cancer models, revealing distinct genetic and microenvironmental influences on immune remodeling. LLC1 tumors promoted an immunosuppressive landscape characterized by B cell expansion, T cell dysfunction, and inflammatory stress responses, whereas Kras^LA2^ tumors maintained immune balance but exhibited metabolic reprogramming and T cell exhaustion. The discovery of Cd3^+^ macrophages might add a novel dimension to our understanding of immune diversity in lung cancer. Together, these findings highlight how tumor genetics drive divergent immune adaptations and identify key cell type-specific pathways that may serve as targets for tailored immunotherapies in non-small cell lung cancer.

## Data Availability

The datasets for this study can be found under the following links: https://bioinformatics.mpi-bn.mpg.de/dizdarevic-et-al-2025, https://bioinformatics.mpi-bn.mpg.de/dizdarevic-et-al-2025-b-cells, https://bioinformatics.mpi-bn.mpg.de/dizdarevic-et-al-2025-t-cells, https://bioinformatics.mpi-bn.mpg.de/dizdarevic-et-al-2025-macrophages, https://bioinformatics.mpi-bn.mpg.de/dizdarevic-et-al-2025-nk-cells.
