# Supplementary material for: Divergent CD45+ immune landscapes shape the lung tumor microenvironment
Source: Front Immunol. 2026 Feb 9;17:1765833. doi: 10.3389/fimmu.2026.1765833 (PMC12926470; doi:10.3389/fimmu.2026.1765833)
Supplement: Supplementary file 1 [file DataSheet1.pdf]

Supplementary Material

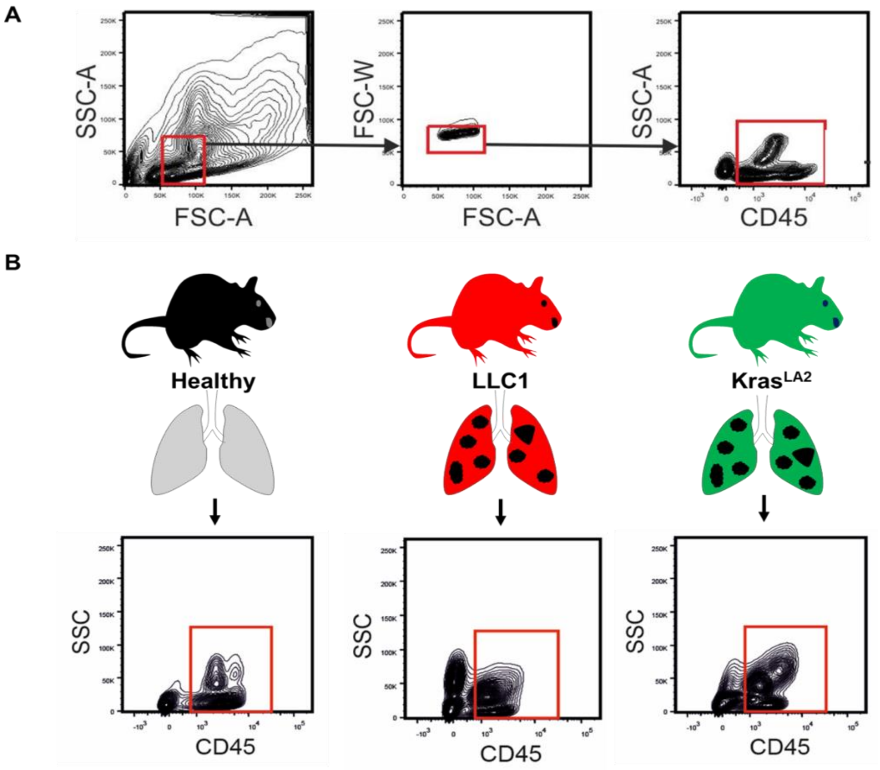

Figure S1. FACS gating strategy for CD45-positive cell sorting from mice lung single cell suspensions. (A) Single-cell suspensions from mouse lungs were analyzed by flow cytometry. Initial gating was performed on FSC-A vs SSC-A to identify cells and exclude debris. To remove doublets, events were sequentially gated on FSC-A vs FSC-W, retaining singlets. Hematopoietic cells were subsequently identified as $CD45^+$ and collected for downstream analyses. **(B)** Schematic diagram of $CD45^+$ cell isolation from the healthy, LLC1, and Kras lung tumor models.

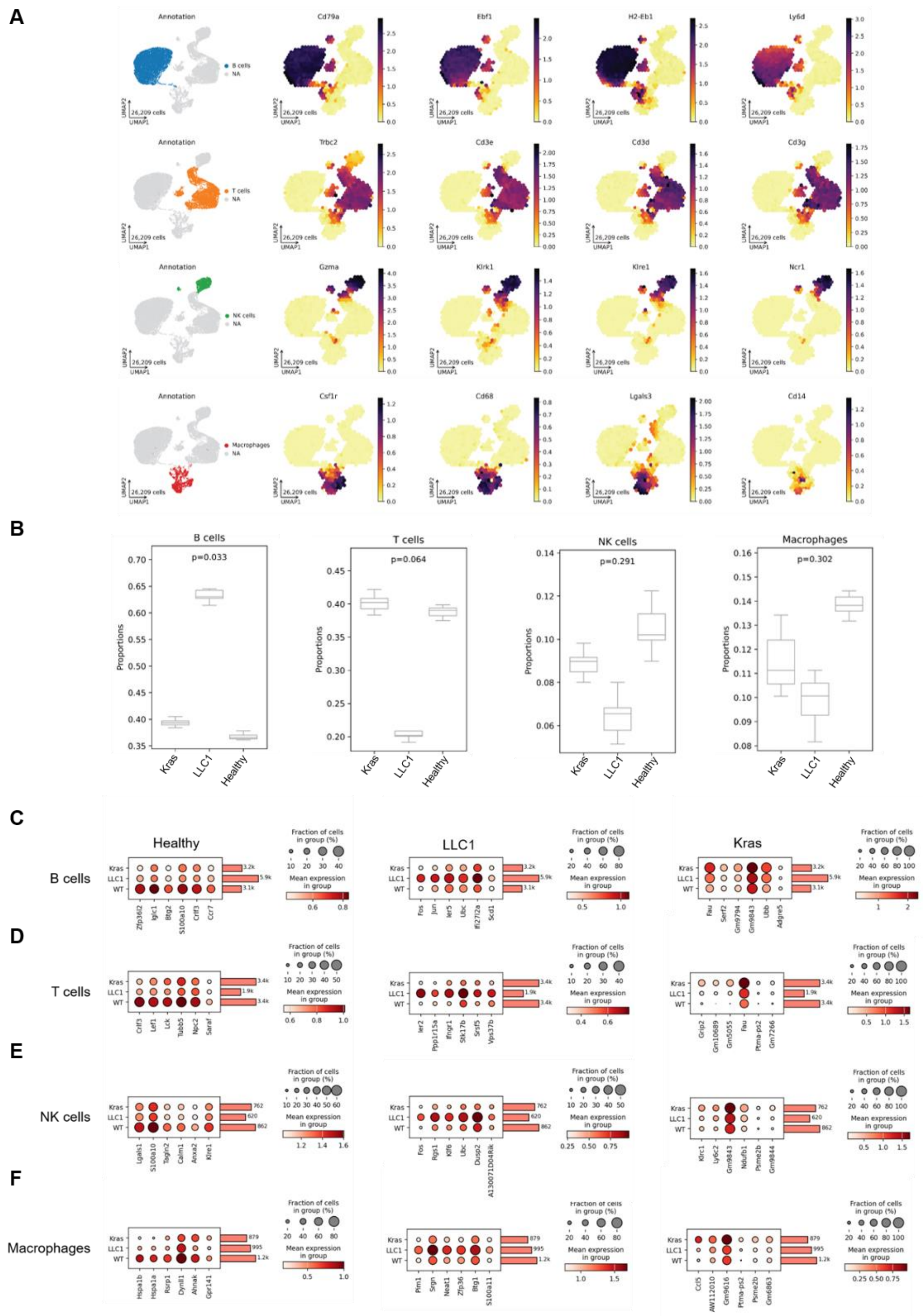

Figure S2. Tumor-specific remodeling of distinct immune cell populations in healthy, LLC1, and Kras lungs (A) UMAP plots illustrate subcluster annotations and marker gene expression for subcluster definition. **(B)** Proportion analysis of subcluster distribution between healthy, LLC1 and Kras tumor bearing mice. Relative changes in immune cell proportions across conditions were analyzed using logit-transformed data. Statistical significance was determined by one-way ANOVA. $P < 0.05$ was considered statistically significant. **(C-F)** Dot plots of B cells **(C)**, T cells **(D)**, NK cells **(E),** and macrophages **(F)** illustrating 6 distinct marker genes in healthy, LLC1 and Kras mouse models. Dot size represents the proportion of cells expressing the gene, and dot color indicates the average expression level within the cluster (scaled expression values).

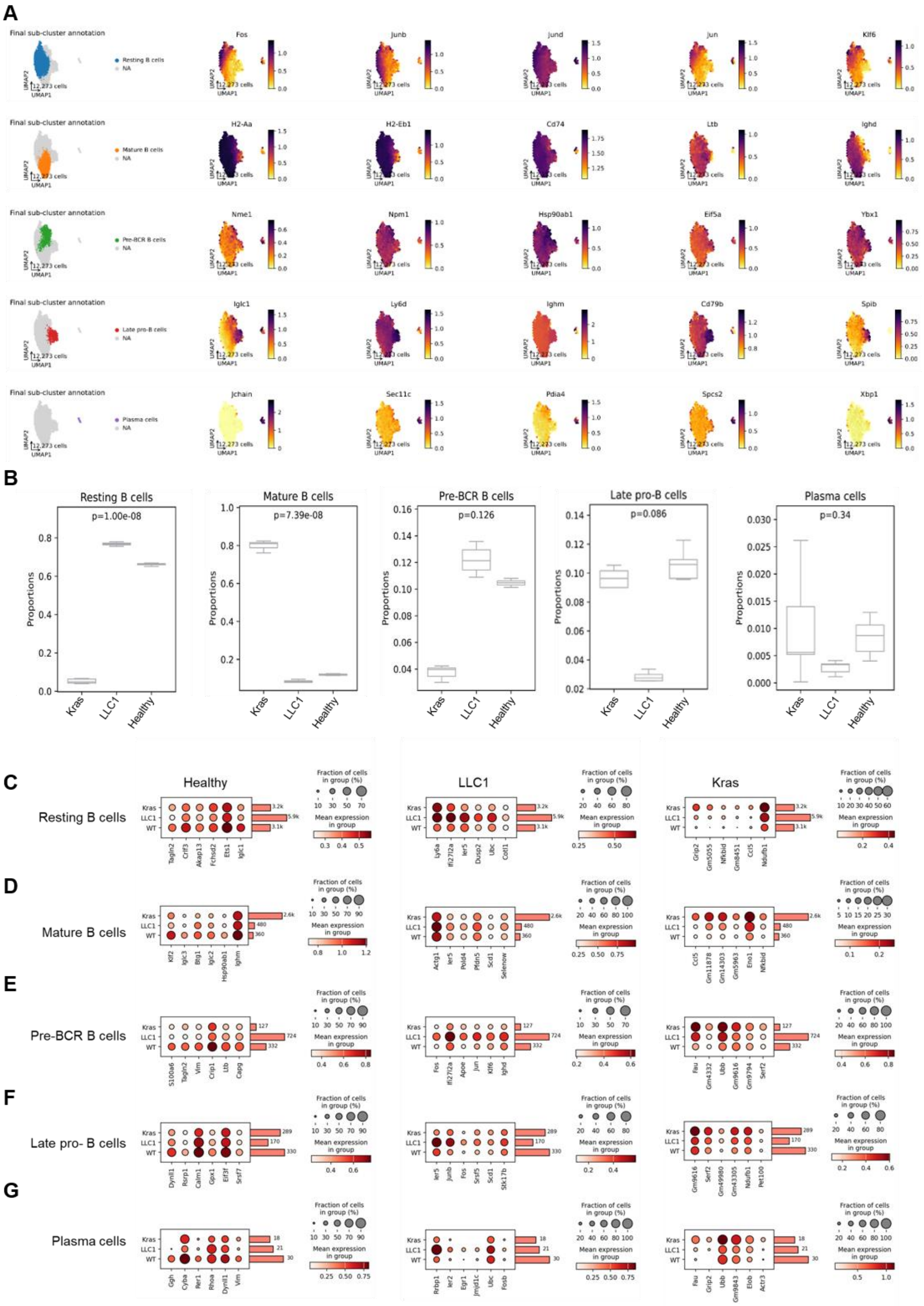

Figure S3. Tumor-specific remodeling of B cell subclusters in healthy, LLC1, and Kras lungs (A) UMAP plots illustrate B cell subcluster annotations and marker gene expression for subcluster definition. **(B)** Proportion analysis of subcluster distribution between healthy, LLC1 and Kras tumor bearing mice. Relative changes in immune cell proportions across conditions were analyzed using logit-transformed data. Statistical significance was determined by one-way ANOVA. $P < 0.05$ was considered statistically significant. **(C-G)** Dot plots of late pro-B cells **(C)**, mature B cells **(D)**, plasma cells **(E),** pre-Bcr B cells **(F)** and resting B cells **(G)** illustrating 6 distinct marker genes in healthy, LLC1 and Kras mouse models. Dot size represents the proportion of cells expressing the gene, and dot color indicates the average expression level within the cluster (scaled expression values).

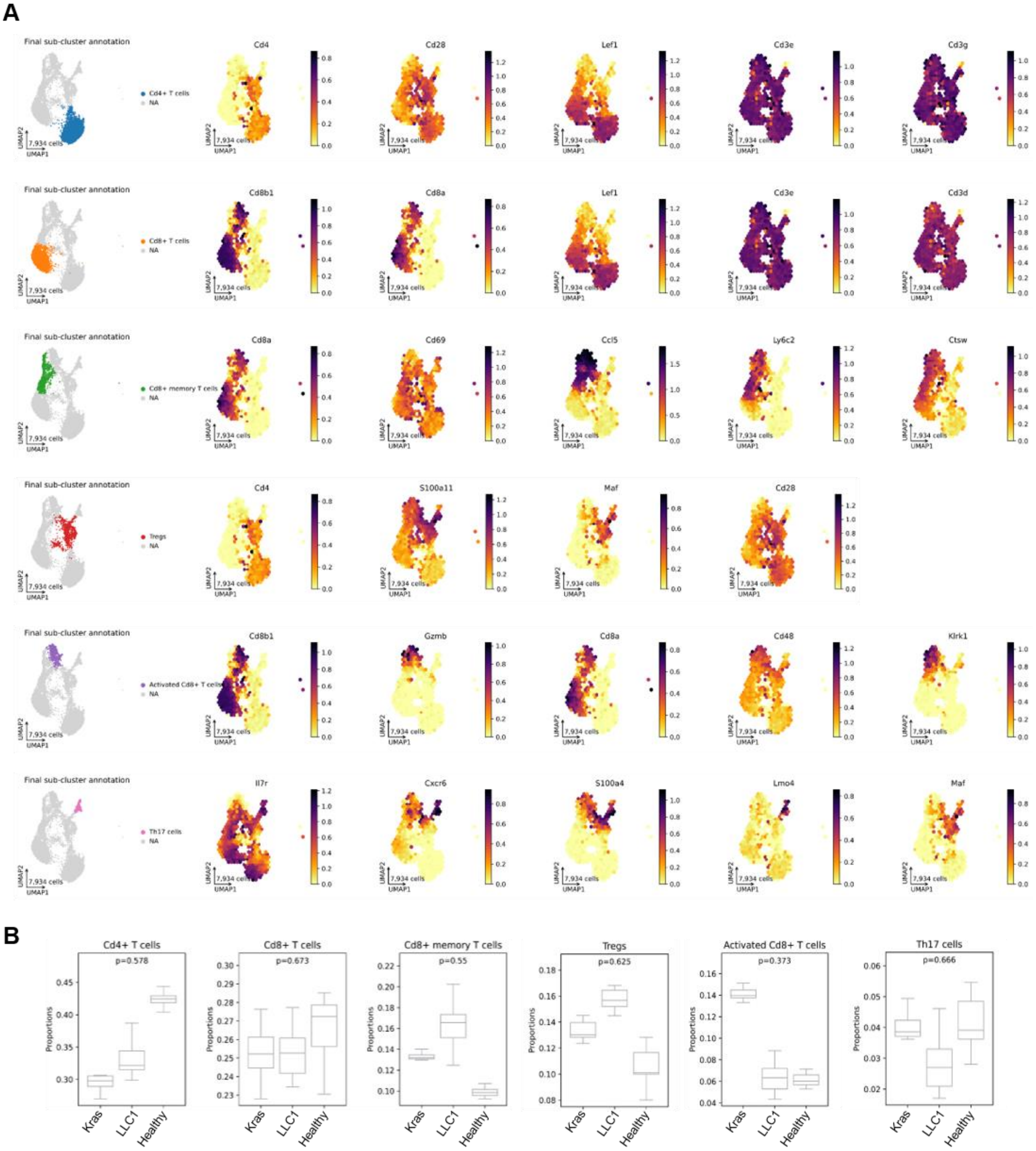

Figure S4. Tumor-specific remodeling of T cell subclusters in healthy, LLC1, and Kras lungs (A) UMAP plots illustrate T cell subcluster annotations and marker gene expression for subcluster definition. **(B)** Proportion analysis of subcluster distribution between healthy, LLC1 and Kras tumor bearing mice. Relative changes in immune cell proportions across conditions were analyzed using logit-transformed data. Statistical significance was determined by one-way ANOVA. $P < 0.05$ was considered statistically significant.

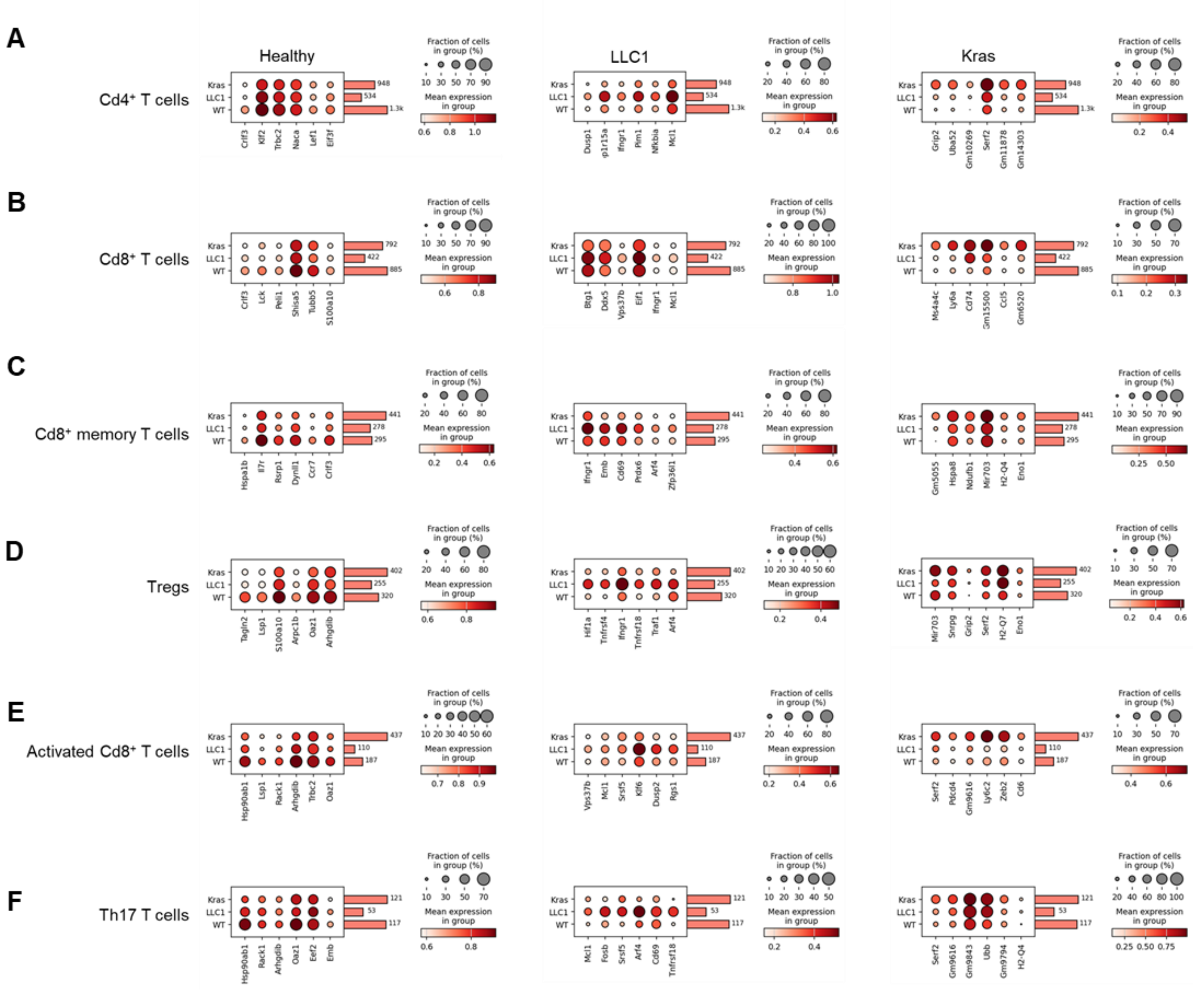

Figure S5. Dot plots of marker genes for T cell subclusters. Dot plots depict marker genes associated with each T cell subcluster. Panels show Cd4^{+} T cells **(A)**, Cd8^{+} T cells **(B)**, Cd8^{+} memory T cells **(C)**, Tregs **(D)**, activated Cd8^{+} T cells **(E)**, and Th17 cells **(F)**, illustrating six distinct T cell phenotypes across healthy, LLC1, and Kras mouse models. For each gene, dot size represents the proportion of cells expressing the gene, and dot color indicates the average expression level within the corresponding cluster (scaled expression values).

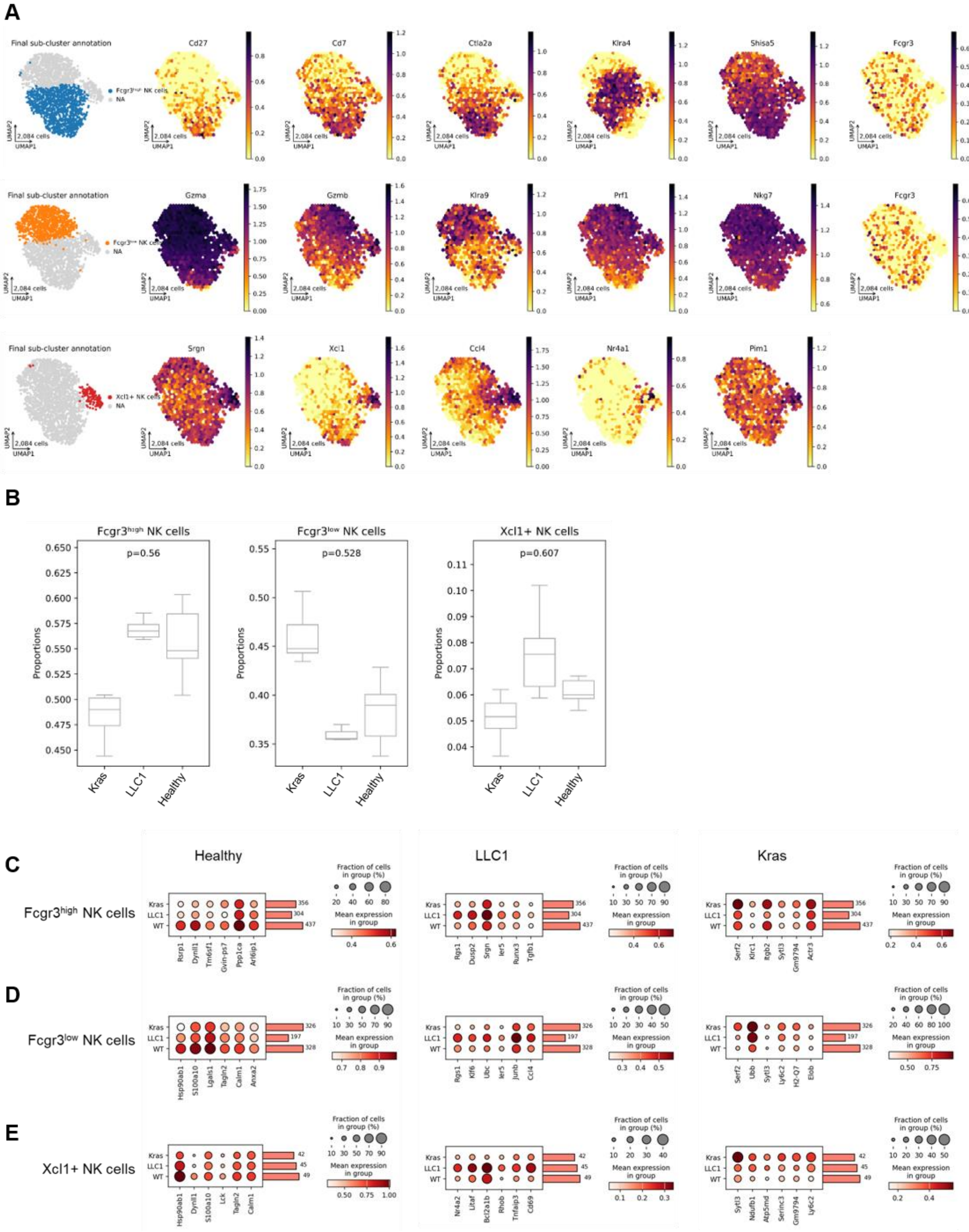

Figure S6. Tumor-specific remodeling of NK cell subclusters in healthy, LLC1, and Kras lungs. (A) UMAP plots illustrate NK cell subcluster annotations and marker gene expression for subcluster definition. **(B)** Proportion analysis of subcluster distribution between healthy, LLC1 and Kras tumor bearing mice. Relative changes in immune cell proportions across conditions were analyzed using logit-transformed data. Statistical significance was determined by one-way ANOVA. $P < 0.05$ was considered statistically significant. **(C-E)** Dot plots of Fcgr3^{high} cells **(C)**, Fcgr3^{low} cells **(D)**, and Xcl1^{+} cells **(E)** illustrating 6 distinct marker genes in healthy, LLC1 and Kras mouse models. Dot size represents the proportion of cells expressing the gene, and dot color indicates the average expression level within the cluster (scaled expression values).

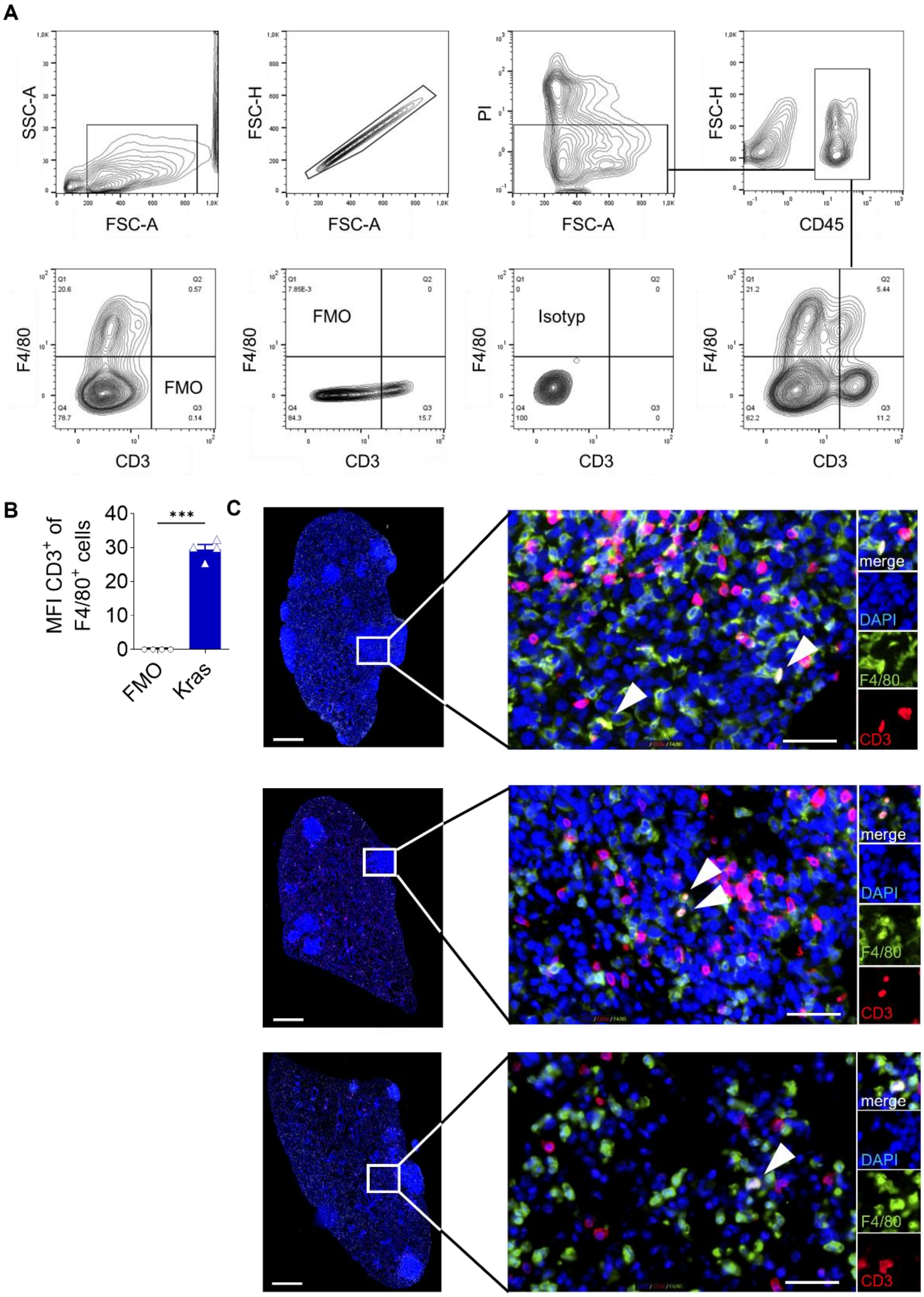

Figure S7. Validation of CD3 and F4/80 coexpressing cells in mice lung tissue. (A) Flow cytometric gating strategy for identification of F4/80⁺CD3⁺ cells from lung single-cell suspensions. Isotype controls and fluorescence-minus-one (FMO) controls were used to define positive gates. **(B)** Mean fluorescence intensity (MFI) of CD3 expression within the F4/80⁺ population in Kras mice compared with FMO controls (n=4). **(C)** Representative immunofluorescence staining of lung tissue from tumor-bearing mice showing costaining with CD3 (red) and F4/80 (green); nuclei were counterstained with DAPI (blue). Scale bars: 2 mm (overview) and 200 µm (zoom). Data are shown as mean ± standard error of the mean using student's t-test. P-values ≤ 0.05 were considered statistically significant for all analyses, ***p ≤ 0.001.

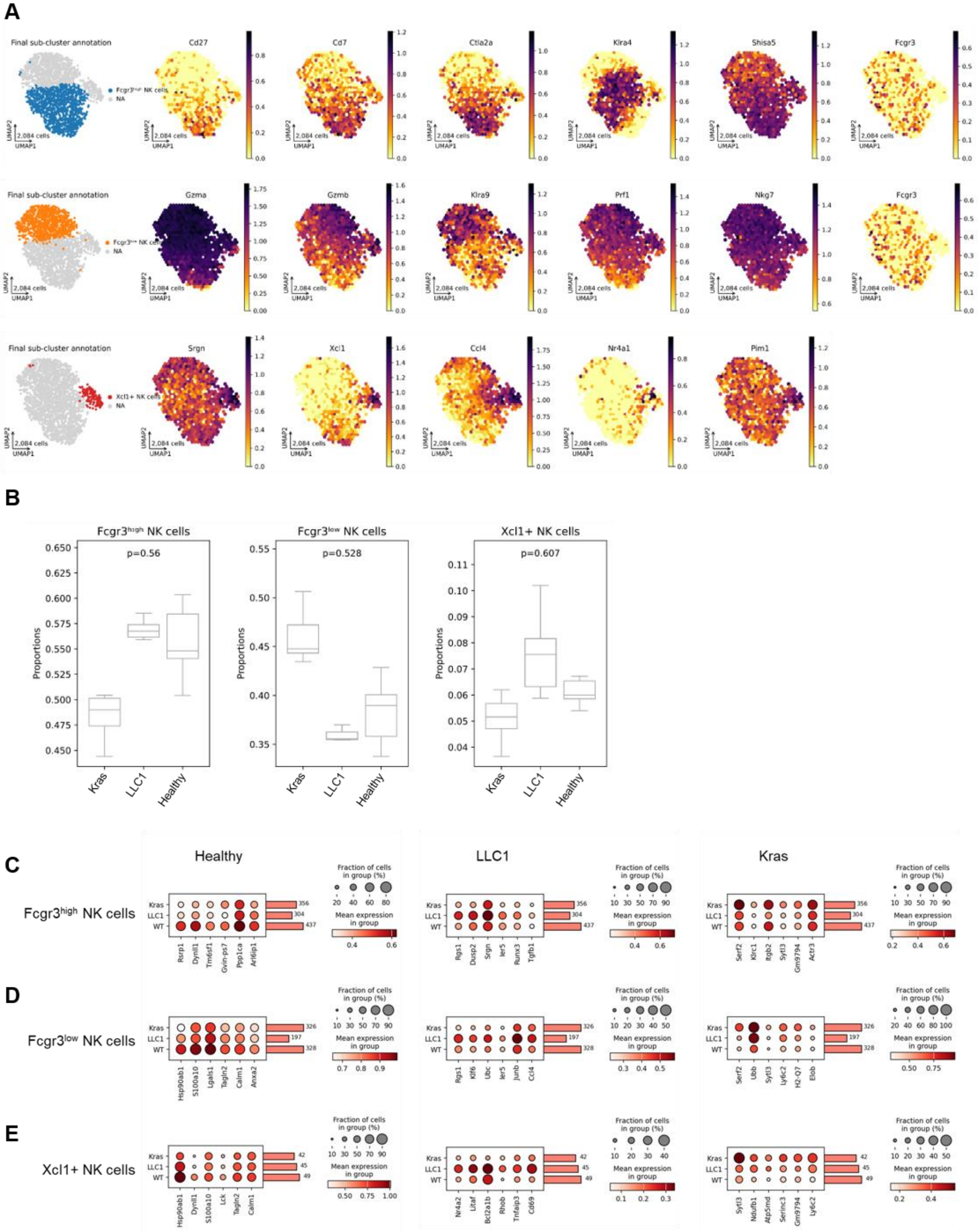

Figure S8. Tumor-specific remodeling of macrophage subclusters in healthy, LLC1, and Kras lungs (A) UMAP plots illustrate Macrophages subcluster annotations and marker gene expression for subcluster definition. **(B)** Proportion analysis of subcluster distribution between healthy, LLC1 and Kras tumor bearing mice. Relative changes in immune cell proportions across conditions were analyzed using logit-transformed data. Statistical significance was determined by one-way ANOVA. $P < 0.05$ was considered statistically significant.

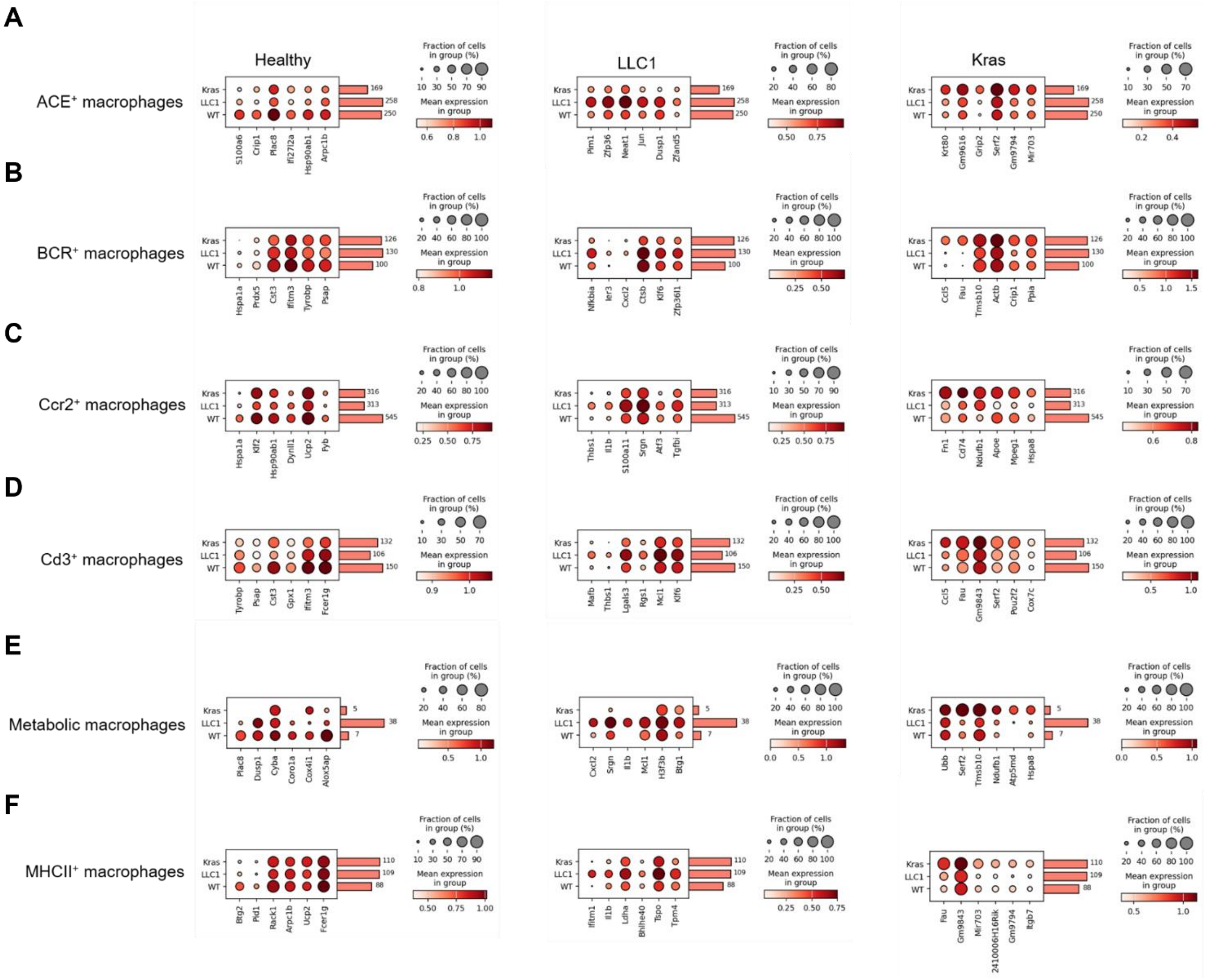

Figure S9. Dot plots of marker genes for macrophage subclusters. Dot plots depict marker genes associated with each macrophage subcluster. Panels show Ccr2⁺ macrophages **(A)**, Ace⁺ macrophages **(B)**, Bcr⁺ macrophages **(C)**, Cd3⁺ macrophages **(D)**, metabolic macrophages **(E)**, and MHCII⁺ macrophages **(F)**, illustrating six distinct macrophage phenotypes across healthy, LLC1, and Kras mouse models. For each gene, dot size represents the proportion of cells expressing the gene, and dot color indicates the average expression level within the corresponding cluster (scaled expression values).

Data availability statement

The datasets for this study can be found under the following links: https://bioinformatics.mpi-bn.mpg.de/dizdarevic-et-al-2025 https://bioinformatics.mpi-bn.mpg.de/dizdarevic-et-al-2025-b-cells https://bioinformatics.mpi-bn.mpg.de/dizdarevic-et-al-2025-t-cells https://bioinformatics.mpi-bn.mpg.de/dizdarevic-et-al-2025-macrophages https://bioinformatics.mpi-bn.mpg.de/dizdarevic-et-al-2025-nk-cells.
